# Phase-wise evaluation and optimization of non-pharmaceutical interventions to contain the COVID-19 pandemic in the U.S.

**DOI:** 10.3389/fpubh.2023.1198973

**Published:** 2023-08-03

**Authors:** Xiao Zhou, Xiaohu Zhang, Paolo Santi, Carlo Ratti

**Affiliations:** ^1^Gaoling School of Artificial Intelligence, Renmin University of China, Beijing, China; ^2^Senseable City Laboratory, Massachusetts Institute of Technology, Cambridge, MA, United States; ^3^Department of Urban Planning, The University of Hong Kong, Hong Kong, Hong Kong SAR, China; ^4^Istituto di Informatica e Telematica del CNR, Pisa, Italy

**Keywords:** non-pharmaceutical interventions, COVID-19 pandemic, public health policymaking, multi-objective optimization, human mobility, multivariate time series analysis

## Abstract

Given that the effectiveness of COVID-19 vaccines and other therapies is greatly limited by the continuously emerging variants, non-pharmaceutical interventions have been adopted as primary control strategies in the global fight against the COVID-19 pandemic. However, implementing strict interventions over extended periods of time is inevitably hurting the economy. Many countries are faced with the dilemma of how to take appropriate policy actions for socio-economic recovery while curbing the further spread of COVID-19. With an aim to solve this multi-objective decision-making problem, we investigate the underlying temporal dynamics and associations between policies, mobility patterns, and virus transmission through vector autoregressive models and the Toda-Yamamoto Granger causality test. Our findings reveal the presence of temporal lagged effects and Granger causality relationships among various transmission and human mobility variables. We further assess the effectiveness of existing COVID-19 control measures and explore potential optimal strategies that strike a balance between public health and socio-economic recovery for individual states in the U.S. by employing the Pareto optimality and genetic algorithms. The results highlight the joint power of the state of emergency declaration, wearing face masks, and the closure of bars, and emphasize the necessity of pursuing tailor-made strategies for different states and phases of epidemiological transmission. Our framework enables policymakers to create more refined designs of COVID-19 strategies and can be extended to other countries regarding best practices in pandemic response.

## 1. Introduction

Since the coronavirus disease 2019 (COVID-19) was initially detected in December 2019, a novel coronavirus designated as the severe acute respiratory syndrome coronavirus 2 (SARS-CoV-2) has rapidly spread across the world and led to a global pandemic (1). Despite increasingly more people getting vaccinated every single day, the world is still struggling to combat the emerging more contagious COVID-19 variants and has witnessed wave after wave of the pandemic here and there. To contain the COVID-19 pandemic, non-pharmaceutical interventions (NPIs) have been widely used as key weapons in some countries that were impacted heavily early on with satisfying effects (2). For instance, the world's first stringent COVID-19 lockdown sparked in Wuhan, the original epicenter of the pandemic. Specifically, China implemented a 76-day travel ban to and from the city and initiated nationally coordinated measures to address the pandemic's impact until the first wave of the pandemic was effectively contained in the country (3). Singapore is another country that adopted aggressive strategies and maintained a low casualty rate (0.15%) compared to the global average (1.38%) (4).

Besides making striking achievements in real-world settings, the effectiveness of COVID-19 NPIs has also been extensively studied in the literature. Among them, a large body of research was targeted at the epidemiological implications of NPIs. Utilizing compartmental models, scholars simulated and predicted the effects of NPIs on multiple epidemic indicators, including infections (2, 3, 5–10), deaths (2, 7–9, 11), the reproduction number (2, 3, 8, 12), and demand for hospital services (5, 9). Touching on similar themes, another line of research focused more on quantifying the effects of NPIs on mobility (13–15) and explored the relationship between human movements and COVID-19 transmission (6, 16–18). Apart from characterizing the unfolding of the pandemic from an epidemiological perspective, there are also studies investigating the impact of NPIs from various angles covering economic contraction (19–22), social issues (23, 24), and mental health (25–28). Adopting a multi-objective perspective, some studies have concentrated on optimizing the socio-economic cost-effectiveness of pandemic mitigation strategies in different countries (29–31). However, these studies did not address the question from the perspective of human mobility.

Some key findings derived from the current state of knowledge can be summarized as follows: (1) Implementing appropriate NPIs is crucial in curbing the spread of the virus (2, 17, 18); (2) The effectiveness of individual interventions alone is often limited, necessitating the combined use of multiple NPIs (7, 32–34); (3) The impact of NPIs on disease burden exhibits nonlinearity (35); (4) Fixed NPI strategies may be less effective compared to those with time-varying adjustment mechanisms (36); and (5) There is an interplay between NPIs and vaccination strategies, particularly concerning highly transmissible variants of concern. Hence, optimizing and tailoring NPIs holds strong potential for reducing both transmission and the clinical burden of the disease.

The encouraging precedents and research basis above demonstrate the role that NPIs can play in controlling the COVID-19 and offer valuable references for other affected regions to handle the spiraling outbreak. However, as time goes by, the situation has become further complicated and highlighted some limitations of the current literature. First, the emphasis of many previous studies is on the pandemic simulation modeling of COVID-19 for more accurate forecasts without a thorough discussion of the relationships between NPIs, human mobility, and virus transmission that are deeply intertwined. In addition, given that the core concern of handling the ongoing COVID-19 pandemic has shifted from containing its spread to restoring the social and economic order in the new normal from a long-term perspective, particular attention should be paid to multi-objective thinking in the design of policy strategies. Moreover, most of the above-mentioned successes were achieved by imposing harsh measures at the beginning of the pandemic, still, opinions are divided sharply on how to devise optimal policy frameworks of NPIs when the population are partly vaccinated against the COVID-19 among different countries.

To contribute in this direction, we choose the U.S. as the focus in the study due to its decentralized decision-making system, which leads to the implementation and enforcement of NPIs highly variable in both time and space across the country with various circumstances for discussion. Using the COVID-19 policy dataset along with epidemiological and mobility data aggregated at the state level, we propose a methodological framework to quantify the underlying relationships between mobility and key measures of population-level transmission in the context of the COVID-19 pandemic both before and after the vaccines became available. We further evaluate the effectiveness of the current combinations of NPIs applied in each state and identify new optimal strategies that can well balance the public health and socio-economic impacts in the fight against COVID-19. Finally, we investigate the similarities and differences across states and phases to provide new insights into the spatio-temporal dynamics for pandemic control. The results of this study provide valuable insights for policymakers to gain a deeper understanding of the ever-changing landscape of the pandemic, enabling them to identify more effective and adaptable solutions that can respond to evolving needs and circumstances.

## 2. Materials and methods

### 2.1. Datasets and preliminary observations

**Figure 1:**
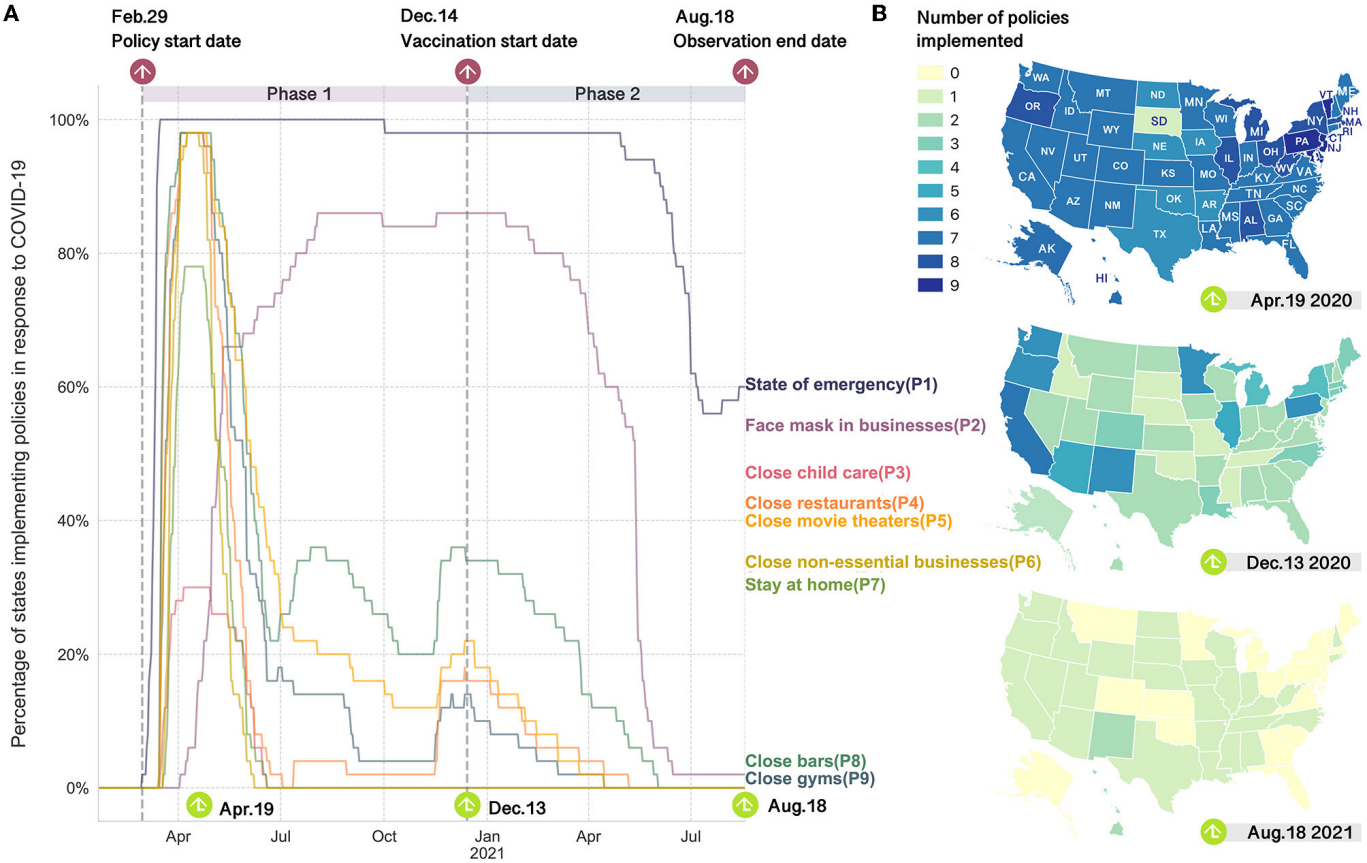
Implementation of anti-contagion policies in response to the COVID-19 over time and space in the United States. **(A)** Percentage of states with the COVID-19 policies being enacted over time. Two vertical dashed lines represent the start dates of COVID-19 policy deployment and vaccine distribution, respectively. **(B)** Maps of number of NPIs implemented by the states on three representative dates. April 19, 2020 represents the period with the most stringent lockdowns. December 13, 2020 is the day before COVID-19 vaccines were administered in the U.S.. August 18, 2021 is the last day of the observation period.

To gain a comprehensive understanding of each state government's response to COVID-19, we utilize the COVID-19 U.S. State Policy Database (37). This database enables us to consider nine prominent NPIs, namely: Declaration of a state of emergency (P1), Mandatory face mask usage in workplaces (P2), Closure of child care facilities (P3), Closure of restaurants (P4), Closure of movie theaters (P5), Closure of non-essential businesses (P6), Implementation of stay-at-home orders (P7), Closure of bars (P8), and Closure of gyms (P9). The temporal and geographical adoption of these policy responses across U.S. states is visually presented in Figure 1. This work covers the study period spanning from February 24, 2020, to August 18, 2021. We further divide the observation period into two main phases according to when the lockdown restrictions and COVID-19 vaccine distribution started in the country. Some general patterns we find include: (1) most states rolled out the strictest lockdown measures in April 2020 to fight against the first wave and gradually rolled back the restrictions as the curve of infections started to flatten; (2) to control the subsequent waves in late June and mid-November 2020, NPIs were reimplemented, however, the number of states participated is much smaller than previously and; (3) since the COVID-19 vaccines were administered, the NPIs were gradually lifted in most states.

Apart from the database of NPIs, we also employ daily COVID-19 case data from the New York Times (38) and aggregated mobility data from Unacast (39) to first draw a comprehensive comparison of the temporal variations in virus transmission, mobility patterns, and policies during the COVID-19 outbreak. Here, two mobility metrics utilized are relative percentage changes in visits to non-essential venues (*VD*) and average travel distance (*TD*) compared to the corresponding day of the week prior to the COVID-19 outbreak for a given date. Two COVID-19 case variables include daily new cases (*NC*) and deaths (*ND*). Specifically, we map the state government policy actions against these variables as well as the instantaneous reproduction number (*R*_*t*_) estimated by the susceptible-exposed-infectious-recovered-susceptible (SEIRS) epidemic model (40) (Supplementary material 3). Here, *R*_*t*_ is a key indicator to monitor the real-time transmissibility of the virus and to estimate the impact of local NPIs on the epidemic. The compartmental model SEIRS is fitted with publicly available COVID-19 case data as mentioned (38). Disease-specific parameters in the model were derived from the recent literature, including a mean incubation period of 5.2 days (95% confidence interval [CI] 4.1–7.0) (41), an average recovery time of 8 days (42) and the mean time to death from the onset of 17.8 days (95% CI 16.9–19.2) (43). In addition, patients are assumed to develop temporal immunity of 180 days, after recovering from the initial infection of the virus (44).

### 2.2. Temporal lagged relationship analysis

Based on the preliminary observations, we further explore multiple temporal dynamic relationships between transmission and mobility variables in a bivariate setting by using vector autoregressive models (45) and the Granger causality tests (46). Here we also employ a sub-period analysis to explore how these variables and the relationships between them changed over time before and after the administration of the COVID-19 vaccines. For clarity, we will take the tests between *R*_*t*_ and *VD* as an example to illustrate in detail how the techniques are employed below.

Vector autoregression (VAR) (45) is a widely used statistical method for multivariate time series analysis. Granger causality analysis (46) was initially developed in econometrics as a technique for investigating the directed interactions between time-series data (47). This statistical concept of causality is based on the prediction that a time series ***x*** (*VD*) Granger causes another time series ***y*** (*R*_*t*_) if the autoregressive forecast of ***y*** can be better explained when the past information from ***x*** is considered (48). After determining the maximum order of integration (*d*) and optimal time lag length (*m*) for *R*_*t*_ and *VD* (Supplementary material 5), we establish bivariate augmented VAR models for the two phases in each state, based on the idea of the Toda-Yamamoto Granger causality test (49) (Supplementary material 6) as follows:


(1) $$\begin{array}{l} y_{t}=\gamma +\overset{m+d}{\underset{i=1}{\sum }}\alpha_{i}y_{t-i}+\overset{m+d}{\underset{i=1}{\sum }}\phi_{i}x_{t-i}+\varepsilon_{t} \end{array}$$

where *y*_*t*_ denotes the value of the time series ***y*** at time *t*, *i* is the length of the lag-time moving window, α_*i*_ and ϕ_*i*_ are the parameters to estimate, ϵ_*t*_ refers to the white noise residual. The variables of ***x*** and ***y*** can be interchanged to test for the Granger causality in the other direction.

### 2.3. Multiple linear regression analysis

Multiple linear regression analysis is then employed to assess the association between policy types and COVID-19 transmission as well as human mobility changes. Specifically, for each state, we build two multiple linear regression models, in which independent variables are policy types [*p*_1_, *p*_2_, …, *p*_*k*_], while the continuous dependent variables are *v*_*t*_ and $R_{t}^{\prime }$, respectively. Here, $R_{t}^{\prime }$ denotes the temporal lagged reproduction number for date *t* with individualized temporal relationships between *v*_*t*_ and *R*_*t*_ in each state. The assumption here is that the changes in human mobility are visible on the same date when the NPIs are issued, while $R_{t}^{\prime }$ is determined by the temporal lagged associations between mobility and the spread of the coronavirus investigated using VAR model with Granger causality test. For instance, since there is a significant Granger causality relationship discovered from *R*_*t*_ to *v*_*t*_ with a lag of *m* = 2 in California during phase 1 (Supplementary Table 5), the corresponding $R_{t}^{\prime }$ for *v*_*t*_ is equal to *R*_*t*−2_. This operation applies to the following two Pareto analysis tasks, the evaluation of existing policy strategies and the design of new policy solutions for each state. To fulfill the second task, the processed data is used to fit multiple linear regression models for each phase in a state as follows:


(2) $$R_{t}^{\prime }=\lambda_{0}+\lambda_{1}p_{1}+\lambda_{2}p_{2}+\cdots +\lambda_{k}p_{k}$$

(3) $$\begin{array}{l} v_{t}=\eta_{0}+\eta_{1}p_{1}+\eta_{2}p_{2}+\cdots +\eta_{k}p_{k} \end{array}$$

where *k* is the number of policy types. The estimated parameters of [λ_0_, λ_1_, λ_2_, …, λ_*k*_] and [η_0_, η_1_, η_2_, …, η_*k*_] are used for the prediction of $\hat{R_{t}}$ and $\hat{v_{t}}$ in the generation process of new optimal policy strategies.

### 2.4. Pareto optimality for COVID-19 policy assessment and design

Next, we elaborate Pareto approaches for the assessment and optimal design of the NPIs in each state. In multi-objective optimization problems, the Pareto-efficient state is achieved if there is no other solution that can bring improvement to one of the objectives without showing degradation in another objective (50). According to this, our optimization problem can be defined as a vector function ***f*** that maps a vector of policy decision variables ***p*** to a tuple of two objectives ***h*** as follows:


(4) $$\begin{array}{l} \text{minimize: }f(p)=min\left\{w_{1}f_{1}(p),w_{2}f_{2}(p)\right\}\\ \text{subject to: }p=(p_{1},p_{2},...,p_{k})\in P\\ \;h=(h_{1},h_{2})\in H \end{array}$$

where *P* is the policy space and *H* is the objective space.

In our case, the optimization goal is to strike a delicate balance between the control of COVID-19 and the recovery of socio-economic vitality, which are indicated specifically by *R*_*t*_ and *VD*, respectively. These two variables are chosen because we consider the estimated *R*_*t*_ a more comprehensive metric to measure the transmissibility of the pandemic and visitation frequency a more appropriate indicator of socio-economic vitality. Based on this assumption, one of the objectives of our research task is to minimize the reproduction number of the virus *f*_*r*_(**p**). In the meanwhile, the value of visitation metric *f*_*v*_(**p**) is expected to be maximized. Accordingly, we set the values of *w*_1_ and *w*_2_ to 1 and -1. Consider two policy decision vectors ***a***, ***b***∈*P*. The policy decision vector ***a*** is said to dominate ***b*** if their objective vectors ***f***(***a***) and ***f***(***b***) satisfy:


(5) $$\begin{array}{l} \;f_{r}(a)⩽f_{r}(b)\wedge f_{v}(a)>f_{v}(b),\\ or\;f_{v}(a)⩾f_{v}(b)\wedge f_{r}(a)<f_{r}(b) \end{array}$$

The set of all the policy decision vectors that are not dominated by any other generates the Pareto optimal set, which provides policymakers with a group of optimal solutions to make a well-informed decision that balances the trade-offs between the public health concerns and socio-economic losses rather than a single-point solution.

In this study, we first use the notion of Pareto optimality to evaluate the performance of existing policy combinations. Specifically, for each given date *t*, we collect the corresponding value of visitation metric *v*_*t*_ and estimate the reproduction number $R_{t}^{\prime }$ using the SEIRS epidemic model with temporal lagged effects considered. These two features collectively form the two-dimensional space, in which the Pareto-optimal set would be explored. For each Pareto-optimal point obtained, we can figure out the corresponding control measures implemented on a particular date for further investigation.

We then explore possible new policy combinations that might be more effective than the current ones by employing the non-dominated sorting genetic algorithm II (NSGA-II) (51) (Supplementary material 8). Specifically, we adopt multiple regression first to estimate the coefficient for each policy type in the prediction of ${\hat{R}}_{t}$ and ${\hat{v}}_{t}$ during different phases with temporal lags effects considered. These parameters calculated are then fed into the NSGA-II algorithm to generate the Pareto optimal solutions. Then the solutions with an estimated ${\hat{R}}_{t}$ no larger than the maximum mean $R_{t}^{\prime }$ of the existing optimal policy strategies are further selected. Different from the implementation of existing policies that are dummy-coded, the parameter estimated through the generic algorithm for each type of policy is a continuous variable, which can be interpreted as the strength of the policy enforcement. According to this, public health policymakers in each state would be able to create more refined designs of COVID-19 strategies with expected effects estimated and make more deliberate decisions regarding what kind of adjustments shall be made to the current schemes and how.

## 3. Results

### 3.1. Temporal associations between COVID-19 transmission, mobility, and state policies

**Figure 2:**
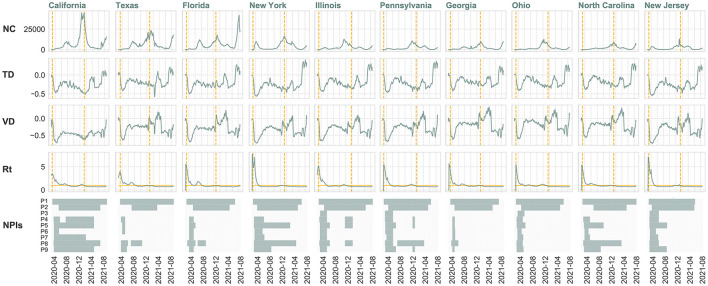
Temporal changes in daily new COVID-19 cases (*NC*), travel distance difference (*TD*), visitation difference (*VD*), instantaneous reproduction number (*R*_*t*_) and policy implementation in the ten states with highest number of confirmed cases from February 24, 2020 to August 18, 2021. 7-day moving average is utilized to smooth volatile case reporting data and human mobility metrics. The start dates of policy implementation and vaccine distribution are indicated by dashed vertical lines. A horizontal line is drawn at *R*_*t*_=1. If *R*_*t*_ is greater than 1, the epidemic is expanding at time *t*, whereas *R*_*t*_ < 1 signals that the epidemic is shrinking.

We first map the state government NPIs against the timelines of mobility and transmission variables to investigate the temporal associations between them. As shown in Figure 2, state authorities typically implemented measures precisely when the *R*_*t*_ value peaked and reinstated NPIs when COVID-19 cases reached alarming levels during subsequent waves. One can also notice that the mobility level seems to be an indicator of how stringent the local NPIs are. In the beginning, when the states adopted the strictest interventions, population mobility experienced a sharp decline from peak to trough in all the states. Then with the adjustments of the NPIs and the application of vaccines, the relative reduction in mobility also changed accordingly. In terms of the relationships between new infections and human mobility, the relevant curves in Figure 2 hint at the existence of time-lagged associations, which will be further investigated through time series analysis next. Due to space limitations, we provide a detailed analysis of California, which has the highest number of COVID-19 cases and a wide range of NPIs. We also highlight key findings for nine other heavily impacted states as examples in the main text. Additional findings for other states in the U.S. can be found in the Supplementary material.

### 3.2. Temporal lagged relationships between mobility and viral transmissibility

Based on the preliminary findings, we further investigate the dynamic temporal lagged relationships between transmission and mobility variables in a bivariate setting using VAR (45) and the Granger causality tests (46). After a series of tests (Supplementary material 5), we then build VAR models with the Toda-Yamamoto Granger causality test according to Equation 1 and display the results in Table 1. As can be observed, the Granger causality relationship from *R*_*t*_ to *VD* is statistically significant at the 1% level during phase 1 in California, but not significant for phase 2. The phenomena can be interpreted as people reacting to the news about confirmed COVID-19 cases by changing their mobility patterns significantly in phase 1. This behavioral response, however, is not that evident after the vaccines were available, hinting at the fact that people felt more protected by vaccines and less keen on constraining their movements. To look in the other direction, it is found that the null hypothesis of no Granger causality from *VD* to *R*_*t*_ can be rejected at the 10% significance level during phase 2, which implies that the visitation change of individuals Granger causes the transmission of the COVID-19 during the period of phase 2.

**Table 1:** Toda-Yamamoto Granger causality test results for transmission and mobility variable pairs in California.

	**Phase 1 (Mar.4 2020–Jan.12 2021)**				**Phase 2 (Jan.13 2021–Aug.18 2021)**			
**Direction**	**Lag(m)**	**Lag(m+d)**	**Chi-square**	**Prob**.	**Lag(m)**	**Lag(m+d)**	**Chi-square**	**Prob**.
*R*_*t*_→***VD***	2	2	16.086^***^	0.000	9	10	11.680	0.307
***VD***→*R*_*t*_	2	2	2.531	0.282	9	10	16.209^*^	0.094
*R*_*t*_→***TD***	2	3	4.544	0.208	8	9	5.687	0.771
***TD***→*R*_*t*_	2	3	1.449	0.694	8	9	5.520	0.787
***NC***→***VD***	9	10	4.565	0.918	8	9	19.714^**^	0.020
***VD***→***NC***	9	10	9.258	0.508	8	9	16.648^*^	0.055
***NC***→***TD***	8	9	18.044^**^	0.035	8	9	7.689	0.566
***TD***→***NC***	8	9	10.722	0.295	8	9	4.660	0.863
***ND***→***VD***	16	17	13.924	0.672	23	24	51.655^***^	0.001
***VD***→***ND***	16	17	15.914	0.530	23	24	46.339^***^	0.004
***ND***→***TD***	23	24	35.936^*^	0.056	20	21	57.509^***^	0.000
***TD***→***ND***	23	24	46.526^***^	0.004	20	21	58.966^***^	0.000

^***^, ^**^, and ^*^ indicate the rejection of the null hypothesis at the 1%, 5% and 10% significance levels, respectively. *NC* represents daily new reported cases. *ND* means daily new deaths. Mobility variables of *TD* and *VD* represent the changes in average travel distance and visits compared to those for the same day of week during non-COVID-19 time period, respectively.

Apart from the variable pair of *R*_*t*_ and *VD*, the Toda and Yamamoto causality test is also performed between other possible pairs of the epidemiological and mobility variables. It can be observed in the table that bi-directional Granger causality emerged between variable pairs of (*ND*, *TD*) in both phases and pair of (*ND*, *VD*) in phase 2. Moreover, all the test results for *ND* during phase 2 reject the null hypothesis at the significance level of 1%. For the corresponding tests between *NC* and the two mobility variables, no Granger causality relationship is found at the 1% significance level. It is also noticeable that the optimal lag lengths selected according to information criteria for *ND* and two mobility metrics are higher than those of *NC* of around one to two weeks, which may correspond to the length of treatment.

### 3.3. Evaluation of existing COVID-19 policy strategies

**Figure 3:**
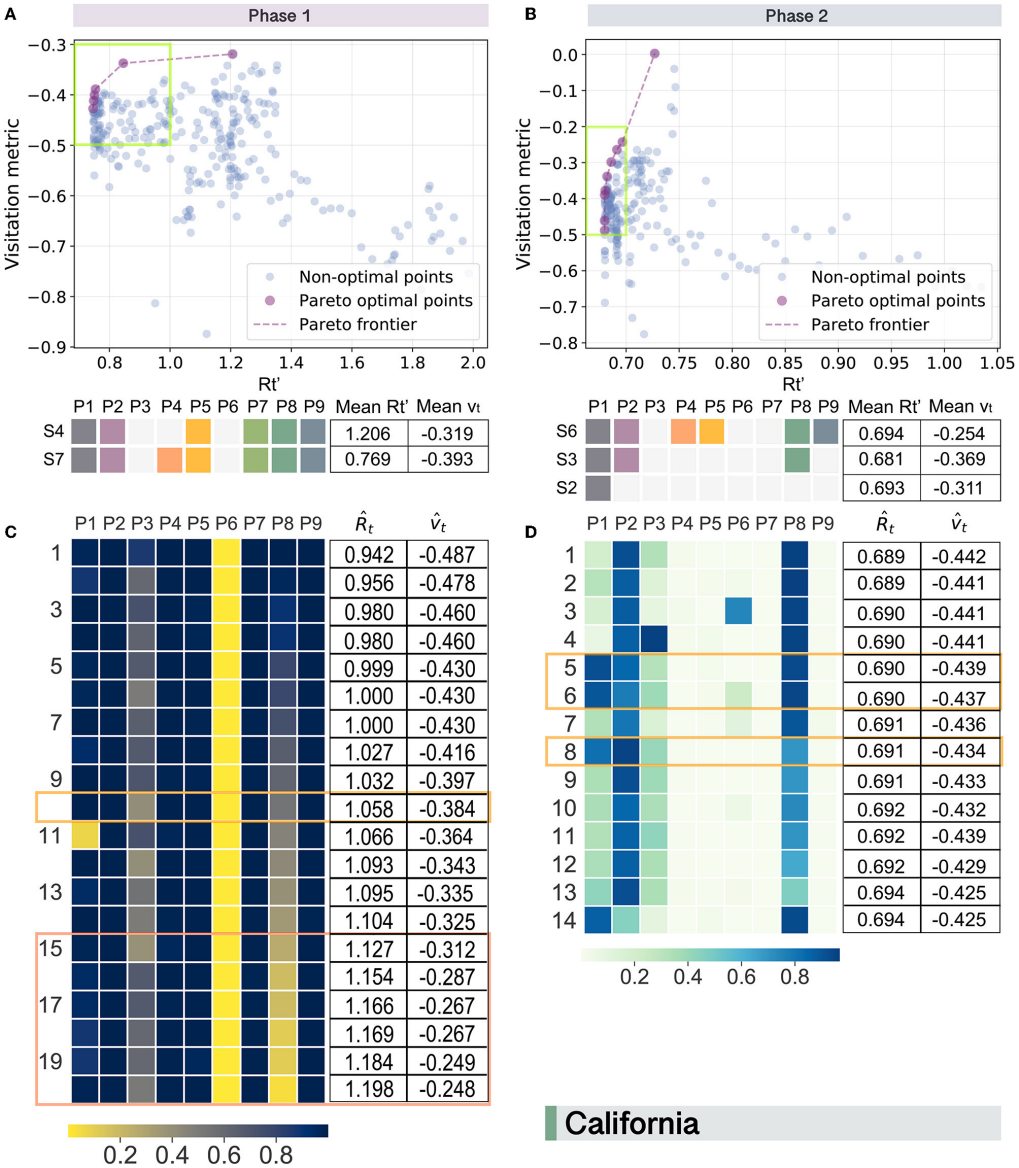
Pareto optimal trade-offs between human mobility (*v*_*t*_) and virus transmission ($R_{t}^{\prime }$) in California during two phases. For existing solutions in phase 1 **(A)** and phase 2 **(B)**, purple-colored spots represent optimal solutions that are connected by dashed lines to visually estimate the Pareto frontier. Candidate points with a value of $R_{t}^{\prime }$ larger than 2 are filtered. Pareto optimal points with a $R_{t}^{\prime }$ between 0.7 and 1 are enclosed by green boxes for phase 1 **(A)** and phase 2 **(B)**, respectively. Corresponding optimal policy strategies after duplicate elimination are displayed in lower sub-figures with average $R_{t}^{\prime }$ and *v*_*t*_ listed in the tables beside them. For potential optimal policy strategies generated for California in phase 1 **(C)** and phase 2 **(D)**, solutions with a ${\hat{R}}_{t}$ less than 1.206 and 0.694 are selected, respectively. Corresponding parameters estimated for the policy types are displayed as heatmaps with predicted ${\hat{R}}_{t}$ and ${\hat{v}}_{t}$ listed in the right tables. Orange rectangle highlights the solutions similar to the existing optimal strategies of S7 and S3; and, coral ones highlight more optimized solutions.

The following offers the results of existing optimal solutions discovered using Pareto approaches in the actual scene. As can be observed from Figures 3A, B, there are in total two and three unique Pareto optimal solutions for California in phase 1 and phase 2, respectively. Among them, the state of emergency (P1) is found to be the only individual policy present in all the optimal solutions. To discuss by phases, it is interesting to observe that the closure of restaurants (P4) is the policy type that distinguishes strategy S4 from S7, connected to a considerable reduction in $R_{t}^{\prime }$, down to 0.769 from 1.206, confirming the relatively high risks of transmitting the virus during convivial activities such as dining in a group. However, closing restaurants also considerably reduces the mobility index, down to -0.393 from -0.319, confirming the trade-off between the need of containing the virus and socio-economic vitality. However, when it came to phase 2, a larger number of enacted control measures did not guarantee a smaller $R_{t}^{\prime }$. Instead, the lowest average $R_{t}^{\prime }$ is generated by a moderate policy strategy S3. In addition, the slope of the Pareto frontier for phase 2 is greater than that for phase 1 in California, implying that the increase in $R_{t}^{\prime }$ is accompanied by a relatively larger recovery of *v*_*t*_ when the vaccines became available.

It should also be noticed that the solutions selected are considered equally good according to the Pareto optimality concept. To decide which solution to choose depends on the policy makers' perspectives about the priority of the two objectives in the optimization task. For instance, if the decision-makers in California intend to relax policies to some extent so that they have as little as possible impact on the normal mobility of the residents in phase 1, the corresponding $R_{t}^{\prime }$ would be as high as 1.206 on average as solution S4 presents.

### 3.4. Design of optimal control strategies for the COVID-19 pandemic

We then explore possible new policy combinations in each state by employing the NSGA-II (51) and present the optimization results for California in Figures 3C, D. Here, the parameter estimated through the generic algorithm for each type of policy can be interpreted as the strength of the policy enforcement that a value closer to 1 represents the strength is relatively stronger, whereas closer to 0 indicates that the implementation of the policy is weaker.

Comparing the optimal solutions generated for phase 1 with those of the existing policy strategies, the last six potential solutions are found more optimized than the current optimal solution S4 since they have both smaller $\hat{R_{t}}$ and larger $\hat{v_{t}}$. This observation suggests that exploring new possible strategies for better trade-offs between virus control and the maintenance of mobility is necessary. Among these six optimal solutions, the NPIs of (P1, P2, P4, P5, P7, P9) are expected to be implemented together with strong strengths. In contrast, the closure of non-essential businesses (P6) seems to be not very necessary, which is in accordance with the phenomenon observed in the evaluation of existing optimal strategies previously. The main distinctions between the potential optimal solutions discovered here from the existing ones for phase 1 lie in the adoption of P3 and P8. Essentially, the new proposed optimal solutions put more emphasis on the importance of closing child care centers (P3) and the flexible adjustment of the shutdown of bars (P8). While for the design of optimal strategies for California in phase 2 (Figure 3D), it presents a significantly different pattern from that for phase 1 (Figure 3C). Specifically, the overall strength of the policies for phase 2 is relatively weaker, and the solutions generated are more heterogeneous. In addition, policymakers in California should also shift the focus of the policy types to wearing face masks in businesses (P2) and closing bars (P8) in the later phase.

**Figure 4:**
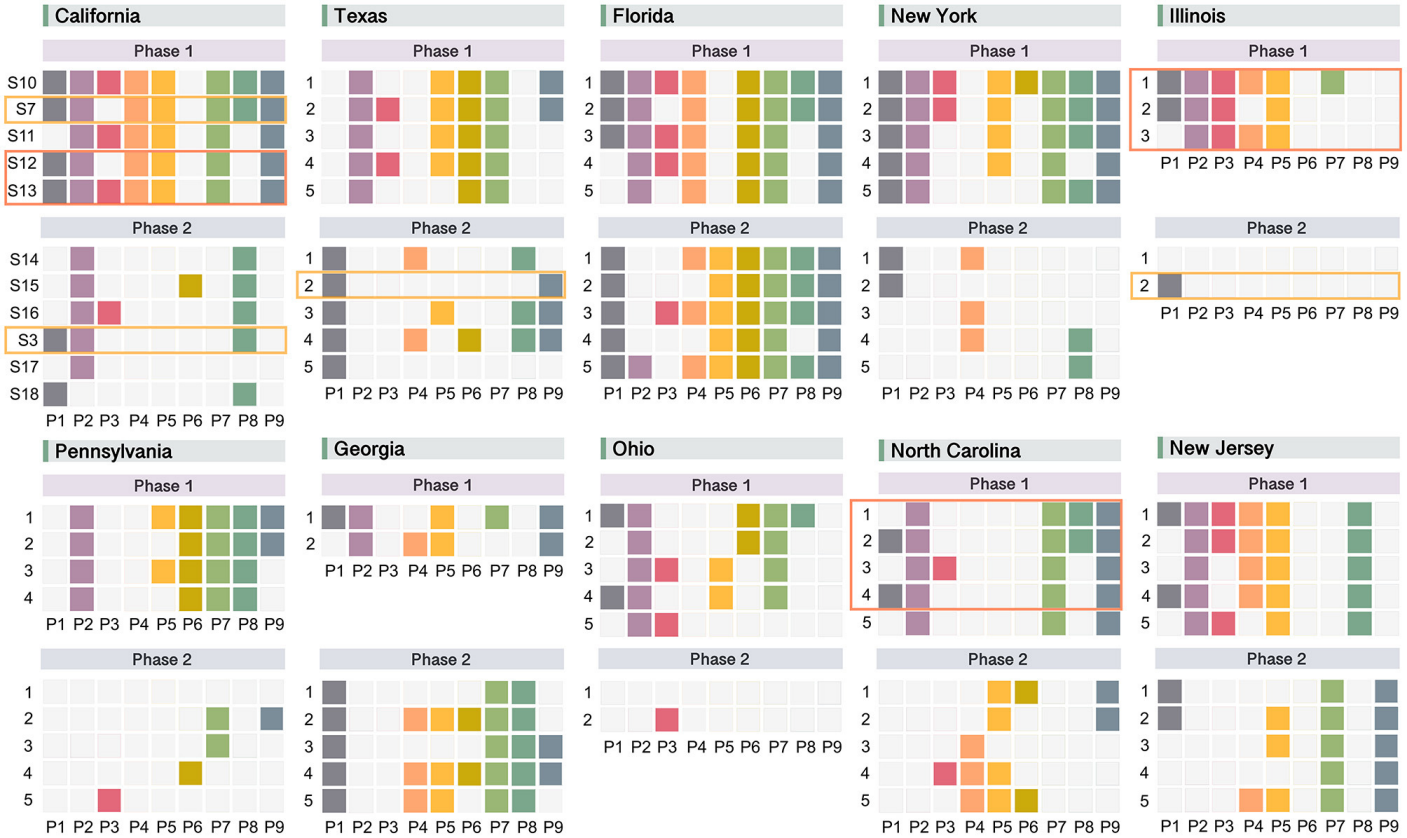
Optimal response strategies generated by NSGA-II for 10 states with the highest number of confirmed COVID-19 cases during different phases. Strategies for each state are listed in ascending order of the average predicted $\hat{R_{t}}$. If more than five optimal solutions are distilled for a certain state and phase, only the top five strategies are retained. Orange rectangles mark solutions already included in the existing strategies for the state; and, coral rectangles highlight more optimized ones.

To provide a clearer picture of how to sketch out the implementation plans, we then replace policy parameters that fall within the ranges (0, 0.5) and (0.5, 1) as 0 and 1, respectively. This simplified version of the optimal policy scheme is shown in Figure 4, from which we can see that there are five and six types of optimal policy strategies extracted for phase 1 and phase 2 in California, respectively. Among them, the current solutions of S7 and S3 are included, while the other nine potential optimal strategies that may achieve better or equally good performance compared to the existing ones offer decision-makers a list of possible alternatives in the fight against COVID-19.

Following the same framework, we conduct analyses for other states and display their simplified optimal strategies in Figure 4. From the view of comparisons across space and time, following major findings emerge: (1) the differences between optimal policy designs for the ten states studied are evident, indicating the necessity of adopting differentiated and tailor-made COVID-19 response strategies in each individual state; (2) existing policy strategies (enclosed by orange rectangles) seldom appear in the list of new proposed ones, suggesting that there are a considerable number of alternatives available for COVID-19 prevention beyond current policy programs; (3) new solutions generated that are more preferable than the current ones (enclosed by coral rectangles with smaller $\hat{R_{t}}$ and higher $\hat{v_{t}}$) show in the first phase of some states (California, Illinois, and North Carolina), offering potential solutions that could be considered to replace the existing ones to policymakers; (4) for each state, two different phases present widely divergent strategies regarding policy types and amount of enacting policies. Specifically, the designs for Phase 2 include fewer policy types and amounts compared to Phase 1 in general, the phenomenon of which may be explained by the availability of vaccines in Phase 2 when the control of COVID-19 no longer relies solely on the NPIs.

## 4. Discussion

This paper introduces a dynamic modeling framework designed to support decision-making processes for local policymakers. Our framework offers flexibility in evaluating and developing refined COVID-19 policy strategies, emphasizing the delicate balance between safeguarding public health and promoting socio-economic vitality. By analyzing shifts in human mobility, we provide insights into effective policy adjustments and decision-making. Here tailor-made adjustment schemes and novel optimal policies in response to the COVID-19 pandemic are generated for state authorities to choose from, depending on their priorities and current vaccination status. This can be achieved through the joint utilization of epidemiological data, mobility data, NPIs, and in-depth exploration of the dynamic relationships between them by fusing multiple techniques.

We started with a discussion of the intertwined associations among the spread of the virus, policy implementation, and human mobility during different phases and discovered some prevailing patterns across the states (Figure 2 and Supplementary Figures 2–5). For instance, the state governments generally began to implement NPIs when the *R*_*t*_ was at its maximum. In addition, human mobility can be used as a proxy for NPIs since it dropped steeply to the lowest level when the most stringent NPIs were issued in the early stages of the pandemic. Then with the subsequential relaxation and tightening of NPIs later on, human mobility levels also changed accordingly. The emergence of COVID-19 vaccines introduced new variables into these interrelationships. Some states (e.g., CA, IL, PA, MI, MN, WA, IA, and OR) began to loosen their NPIs, which coincided with a virus transmission decline and human mobility increase.

Another key observation that warrants further investigation is the existence of underlying temporal associations between human mobility and virus transmission, which might be bidirectional and dynamic. When adjustments were made to NPIs, human mobility offered relatively immediate responses while the corresponding changes in virus transmission would take some extra time to reflect on confirmed cases. Moreover, the temporal relationships between virus transmission and human mobility might be bidirectional and dynamic. For instance, people would adjust their travel behaviors if they saw a dramatic rise in COVID-19 cases; and the changes in mobility trends would, in turn, affect the confirmed cases in a few days. Furthermore, the relationships would also change with the advent of the vaccines. These observations impel us to ponder over the temporal associations between mobility and virus transmission by employing the VAR model and Granger causality analysis in a phased manner for further investigation. For instance, the results of VAR models prove the existence of temporal lagged effects as well as Granger causality relationships between some transmission and mobility variable pairs during different phases. With regard to the variable pair of (*R*_*t*_, *VD*) that we pay particular attention to, significant Granger causality relationships are largely discovered in the states affected the most by COVID-19 in the U.S. (Supplementary Table 7). Here for each state, at least one direction in one phase offers results that are significant at the 5% level. In addition, we observe a relatively stronger Granger causality relationship between *ND* (death count) and mobility patterns compared to that of *NC* (daily infections), which aligns with findings from previous studies (52–54). These studies have highlighted the use of death count as a more reliable metric, considering that the actual number of infected cases is expected to be significantly larger than reported. These findings deepen our understanding of the transmission dynamics of COVID-19 and human behaviors by revealing the intricate temporal associations between them. This knowledge enables more comprehensive policy-making that considers the temporal effects, leading to more effective strategies.

We then evaluate the performance of existing policy strategies across the states using Pareto analysis, so as to offer policymakers a lens for looking back to identify effective policy combinations and adjust less effective ones in the future. For the pre-vaccine stage, to measure the contribution of policy types to health-economic balance individually, state of emergency (P1) and wearing face masks (P2) show as the most important NPIs that they appear in all the optimal strategies among the top ten states (Supplementary Table 8). The closure of recreational services (P8, P5, P9, P4) comes next with moderate impact, while essential businesses (P6) and child care centers (P3) do not present in any of the optimal strategies. From the perspective of the policy mix, the co-activation of P1 and P2 accounts for the largest proportion (30%) and is presented in the optimal strategies for six states (TX, FL, IL, GA, OH, NJ). The second most frequent policy combination is (P1, P2, P8), which accounts for 25% of the optimal strategies. This observation implies the importance of ordering bars to close in the COVID-19 fight when vaccines have not been authorized for use. The reason why the closure of bars plays a more significant role in achieving the optimal status than other types of entertainment venues may be due to the fact that the social atmosphere in bars tends to be more lively, mixed, and closer with a relatively shorter social distance. This finding implies that paying closer attention to the control of gathering sites where social scenes are more complex (e.g., bars) is particularly necessary before the advent of vaccines. Then when the COVID-19 vaccines were available, the percentage of each policy type shown in the optimal strategies decreased in general (Supplementary Table 9). However, from a ranking view, what does not change compared to Phase 1 is that the most effective individual policy types are still P1, P2, and P8. In terms of the policy combinations, there are two most dominant strategies, namely (P1, P2) and enacting P1 only. Both of the strategies account of 27% and present in six states out of the ten.

Finally, we employ the NSGA-II algorithm to generate new optimal policy strategies. Here, the results reveal substantial differences between different phases and states, highlighting the necessity of designing spatio-temporal tailor-made policy strategies. For instance, wearing face masks (P2) is particularly important for all the states in the pre-vaccine period (Supplementary Figures 8–16). However, this is not the case in the later phase. The policy type of closing restaurants (P4) is essential for FL and NJ in phase 1, but not that important for the states of TX, NY, PA, and NC. By considering the state-tailored temporal lag effects in Granger causality tests and regression analyses in a phase-wise manner, we provide policymakers with a spatio-temporal context-aware guiding policy formulation reference. It also enables them to compare the expected effects of potential optimal solutions with the performance of existing strategies and make more deliberate decisions regarding what kind of adjustments shall be made to the current schemes and how. Moreover, the implementation strategies proposed by our framework are more fine-grained than the existing ones. This is reflected in the strengths of policy enforcement as outputs for decision-makers. Furthermore, our framework also offers sophisticated implementation strategies that produce the strengths of policy enforcement as outputs for decision-makers.

Our study has several limitations. First, we mainly focus on NPIs implementation in the regions with the most confirmed cases, since they play a more crucial role in combating the COVID-19 pandemic. However, an in-depth analysis of areas with fewer cases may raise other intriguing questions, such as why they performed relatively better in the fight against COVID-19. This sort of exploration may lead to a deeper understanding of how to improve the combined power of NPIs or offer novel insights into reasons for the effectiveness of control measures varying considerably across regions. This future investigation may require the additional use of demographic data, which can also help reveal the underlying logic of the results we have obtained. In addition, we only consider public health and human mobility as two primary objectives in the optimization of this study. Other socio-economic costs due to COVID-19 pandemic like psychological distress, economic recession, job losses, increased government debt, and social unrest can be discussed in future work. It is also important to note that the specific socio-economic costs can vary across countries and regions, and further research can provide a more comprehensive understanding of the impact of COVID-19 on different aspects of society and the economy. One additional limitation of this study is the absence of specific data on the composition of viruses in each state during the observation period. Consequently, we were unable to account for the variations in virus composition. Instead, our study focuses on comparing the optimized combinations of different policies and their potential effects on various variants. Although the precise timing of these effects may differ across variants, conducting more comprehensive analyses for each variant would be an intriguing avenue for future research.

In conclusion, we provide detailed insights into the spatio-temporal dynamics of the COVID-19 pandemic during different stages and highlight the essential role of some core intervention portfolios in controlling the pandemic. The methodology proposed here can offer policymakers reasoned estimates of the potential effectiveness of the NPIs to be attained and becomes even more critical when health systems are facing extreme loads in the U.S. In addition, the framework presented can be useful for populations without access to local COVID-19 data and for similar scenarios in the future involving emerging infectious diseases where relevant outcomes are not yet available.

## Data availability statement

The original contributions presented in the study are included in the article/Supplementary material, further inquiries can be directed to the corresponding author.

## Author contributions

XZhou, XZhang, and PS conceived the experiments. XZhang collected the pandemic and mobility data. XZhou conducted the experiments and wrote the paper. PS and CR coordinated the project. All authors participated in the discussion of results, provided critical feedback, and reviewed the manuscript. All authors contributed to the article and approved the submitted version.
