# Supplementary material for: Phase-wise evaluation and optimization of non-pharmaceutical interventions to contain the COVID-19 pandemic in the U.S
Source: Front Public Health. 2023 Aug 3;11:1198973. doi: 10.3389/fpubh.2023.1198973 (PMC10434774; doi:10.3389/fpubh.2023.1198973)
Supplement: Supplementary file 1 [file Data_Sheet_1.pdf]

Supplementary Material

1 SUPPLEMENTARY NOTE 1: SUMMARY AND DESCRIPTIONS OF TYPICAL NPIS

In response to the COVID-19 crisis, governments at different levels in the U.S. have adopted various place-based NPIs to reduce physical mobility and social contact. To get an overall picture of each state government's policies related to coronavirus, we employed the COVID-19 U.S. state policy Database collated by the researchers at the Boston University School of Public Health (Raifman et al., 2020). The nine typical policies we considered are listed in Supplementary Table S1.

Label	Policy Name	Description
**P1**	State of emergency	A state issued any type of emergency declaration
**P2**	Face mask in businesses	Mandate face mask use by employees in public-facing businesses
**P3**	Close child care	A state closed day cares statewide
**P4**	Close restaurants	A state closed restaurants (except for take out)
**P5**	Close movie theaters	A state closed movie theaters statewide
**P6**	Close non-essential businesses	A state closed non-essential businesses statewide
**P7**	Stay at home	A state issued statewide stay at home/shelter in place order
**P8**	Close bars	A state closed bars statewide
**P9**	Close gyms	A state closed indoor gyms/fitness centers

Table S1. Summary of typical NPIs considered and corresponding descriptions.

2 SUPPLEMENTARY NOTE 2: SUMMARY STATISTICS FOR VARIABLES IN CA

We display basic statistics of the transmission and mobility variables in California in the table below. R_t experienced a significant decrease from up to 3.469 in phase 1 to below 1 during phase 2. In terms of the mobility changes, a clear pattern emerged is that California suffered relatively greater declines in travel distance and visits during phase 1 compared to its counterpart.

Variables	Phase 1 (Mar.4 2020 - Jan.12 2021)					Phase 2 (Jan.13 2021 - Aug.18 2021)				
	NC	*ND*	R_t	*TD*	*VD*	*NC*	*ND*	R_t	*TD*	*VD*
**Mean**	8934	99	1.255	-0.309	-0.489	6503	157	0.721	-0.217	-0.437
**Min**	10	0	0.746	-0.485	-0.726	883	16	0.680	-0.517	-0.628
**Max**	44768	531	3.469	-0.024	-0.044	39587	561	1.021	0.117	-0.040
**Std.Dev.**	11780.600	90.258	0.588	0.102	0.110	7253.021	169.436	0.062	0.182	0.091

Table S2. Summary statistics for COVID-19 and mobility variables in California during different phases. Phase 1 represents the period when NPIs have been adopted but no vaccines had become available yet, while phase 2 represents the time when the NPIs and vaccines were deployed together. *NC* represents daily new reported cases. *ND* means daily new deaths. Mobility variables of *TD* and *VD* represent the changes in average travel distance and visits compared to those for the same day of week during non-COVID-19 time period, respectively.

3 SUPPLEMENTARY NOTE 3: DESCRIPTION OF THE EPIDEMIOLOGICAL MODEL

We apply a modified SEIRS (Bjørnstad et al., 2020) compartmental model to detect the local spread of COVID-19 in the U.S. at different stages and estimate the effects of containment measures on the epidemic evolution in each state. Supplementary Fig. S1 displays the structure of the SEIRS compartmental model.

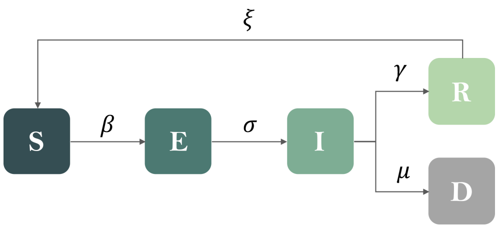

Supplementary Figure S1: The structure of SEIRS epidemic model. The blocks represent the compartments in the model. The arrows represent flows from one compartment to the next and are annotated with transition rates between them. The epidemiological phases include: susceptible (***S***), exposed (***E***), infected (***I***), recovered (***R***), and dead (***D***).

The coupled dynamics of the compartments are governed by the following set of ordinary differential equations:

$$\frac{dS(t)}{dt} = -\frac{\beta S(t)I(t)}{N(t)} + \xi R(t) \tag{S1}$$

$$\frac{dE(t)}{dt} = \frac{\beta S(t)I(t)}{N(t)} - \sigma E(t) \tag{S2}$$

$$\frac{dI(t)}{dt} = \sigma E(t) - (\gamma + \mu)I(t) \tag{S3}$$

$$\frac{dR(t)}{dt} = \gamma I(t) - \xi R(t) \tag{S4}$$

$$\frac{dD(t)}{dt} = \mu I(t) \tag{S5}$$

where β is the rate of infection transmission, which is normalized by $N(t)$, representing the total population at time t: $N(t) = S(t) + E(t) + I(t) + R(t)$. σ denotes the incubation rate of latent individuals becoming symptomatic, and is calculated as the inverse of incubation period length of COVID-19. Similarly, γ represents the recovery rate, the inverse of which is the average time an infected person needs to be recovered. In our model, patients are assumed to develop short-term immunity of $1/\xi$ days after recovering from the viral infection and become susceptible to the virus again. However, their unlucky counterparts who died from the virus at the fatality rate μ would be removed from the transmission process spontaneously.

In compartmental epidemic models, a key parameter used to characterize the transmissibility of the virus is called the basic reproduction number (R_0). It corresponds to the average number of secondary cases

generated by per infectious individual in a fully susceptible population and can be computed from the following equation:

$$R_0 = \frac{\beta}{\gamma + \mu} \tag{S6}$$

While R_0 represents the potential for disease transmission in an idealized scenariois, it is not influenced by NPIs. To monitor the effectiveness of control measures and predicting the future trajectory of an epidemic, R_t representing the effective reproduction number at a specific point in time during an epidemic can be employed. It takes into account the current population immunity level, interventions, and changes in the transmission dynamics over time. Unlike R_0, R_t can vary as the epidemic progresses and is influenced by factors such as public health measures, vaccinations, behavior changes, and variations in the transmission rate. When NPIs are implemented effectively, they can lower R_t below the value of R_0, indicating a reduction in transmission and the potential for controlling the epidemic. The logic behind this is that a drastic reduction in mobility associated with the implementation of COVID-19 containment measures would decrease the contact rate between infected and susceptible individuals. Under this assumption, a sliding window-based extension of the SEIRS model is proposed to capture the real-time epidemic dynamics of coronavirus during different phases of intervention development. Specifically, using a sliding window of size $2n + 1$, we calculate a time-varying transmission rate of the infection at time t as:

$$\beta(t)' = \sum_{i=t-n}^{2n+1} \beta(i)/(2n + 1) \tag{S7}$$

Based on the dynamic contact rate $\beta(t)'$, we then infer the instantaneous reproduction number R_t to track the epidemic progression over time and estimate the impact of local interventions. Here, if R_t is greater than 1, the epidemic is expanding at time t, whereas $R_t < 1$ indicates that the epidemic is shrinking. The expression for the R_t is:

$$R_t = \frac{\beta(t)'}{\gamma + \mu} \tag{S8}$$

It allows a rapid detection of the ongoing evolution during the COVID-19 pandemic, taking the virus's epidemiological characteristics and human mobility patterns that may be significantly affected by local control measures into account. Specifically, we simulate the local spread of the COVID-19 pandemic in different states using the compartmental model introduced above, fitted with publicly available data from the New York Times (NYT, 2021). Disease-specific parameters in the model were derived from the recent literature as listed in TableS3.

**Parameter**	**Value**	**Reference**
σ	5.2 days	Li et al. (2020)
γ	8 days	Maier and Brockmann (2020)
μ	17.8 days	Verity et al. (2020)
ξ	30 days	Seow et al. (2020)

Table S3. Parameters of the SEIRS model.

4 SUPPLEMENTARY NOTE 4: TIME SERIES OF COVID-19 TRANSMISSION, MOBILITY, AND POLICIES

We display the temporal changes of daily new COVID-19 cases, travel distance metric, visitation metric, instantaneous reproduction number R_t, and policy implementation in states with 11th to 50 highest number of confirmed cases from Feb. 24, 2020 to Aug. 18, 2021 in Supplementary Fig. S2-S5. Here 7-day moving average is utilized to smooth volatile case reporting data and human mobility metrics. The start dates of policy implementation and vaccine distribution are indicated by dashed vertical lines.

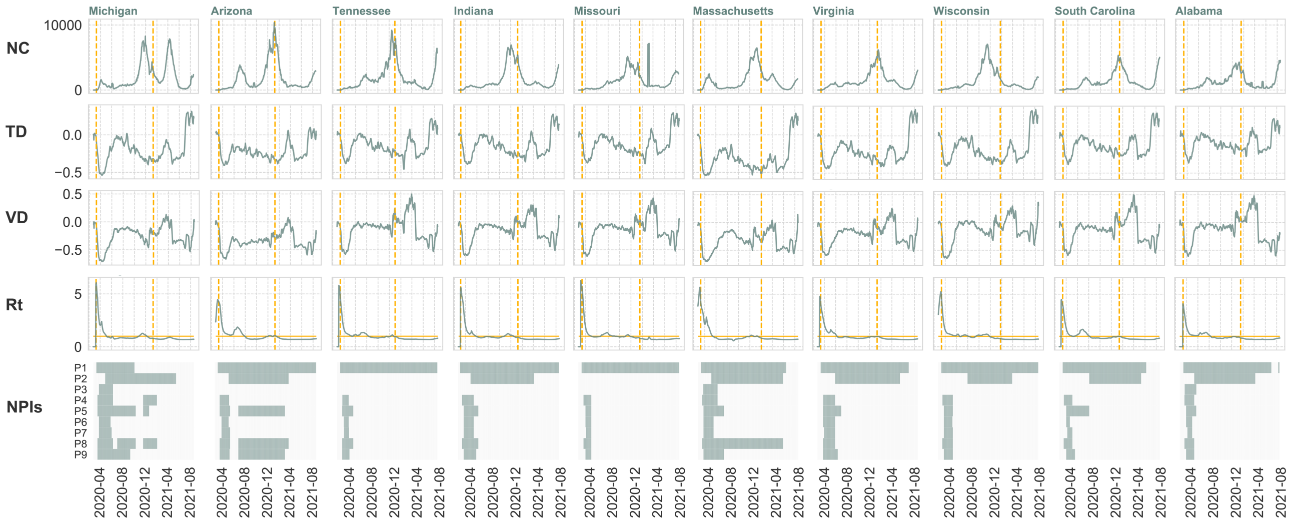

Supplementary Figure S2: Temporal changes in daily new COVID-19 cases (NC), travel distance difference (TD), visitation difference (VD), instantaneous reproduction number (R_t) and policy implementation in the ten states with 11th to 20th highest number of confirmed cases from Feb. 24 2020 to Aug. 18 2021.

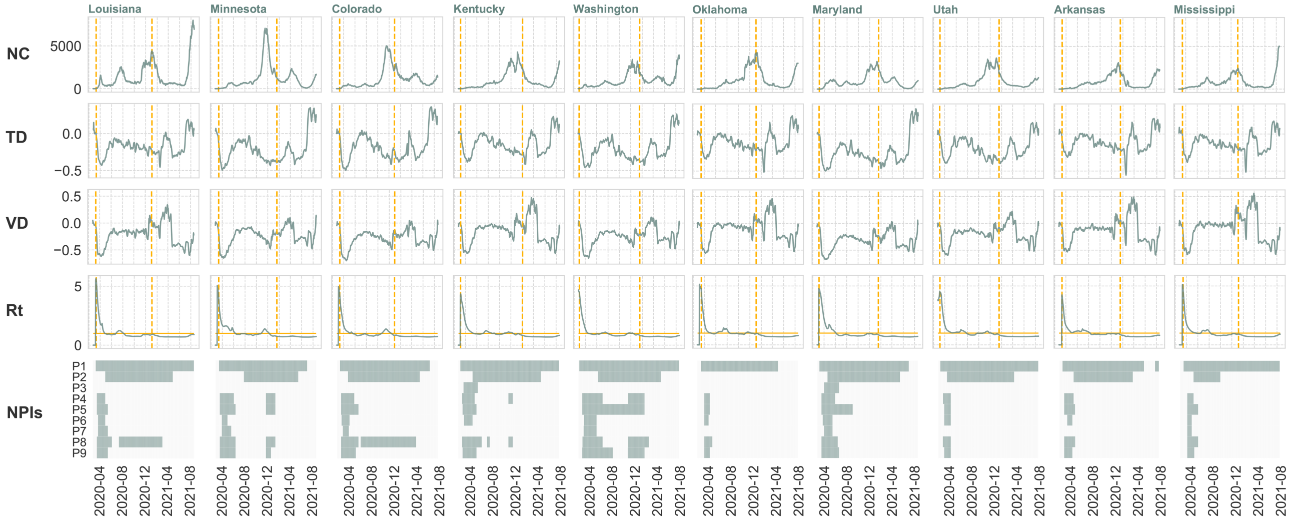

Supplementary Figure S3: Temporal changes in daily new COVID-19 cases (NC), travel distance difference (TD), visitation difference (VD), instantaneous reproduction number (R_t) and policy implementation in the ten states with 21th to 30th highest number of confirmed cases from Feb. 24 2020 to Aug. 18 2021.

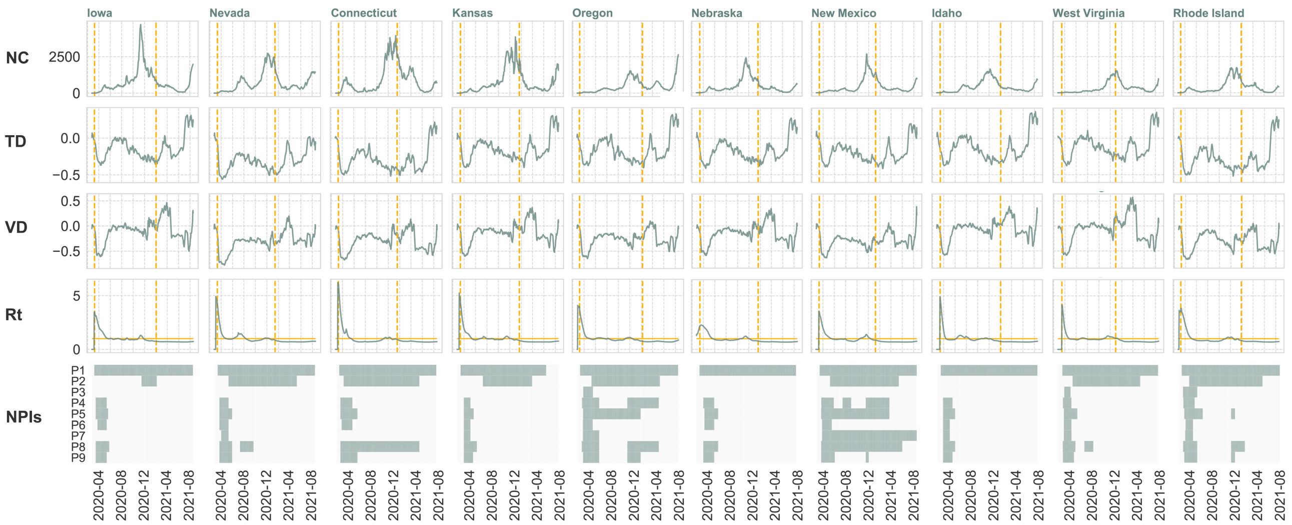

Supplementary Figure S4: Temporal changes in daily new COVID-19 cases (NC), travel distance difference (TD), visitation difference (VD), instantaneous reproduction number (R_t) and policy implementation in the ten states with 31th to 40th highest number of confirmed cases from Feb. 24 2020 to Aug. 18 2021.

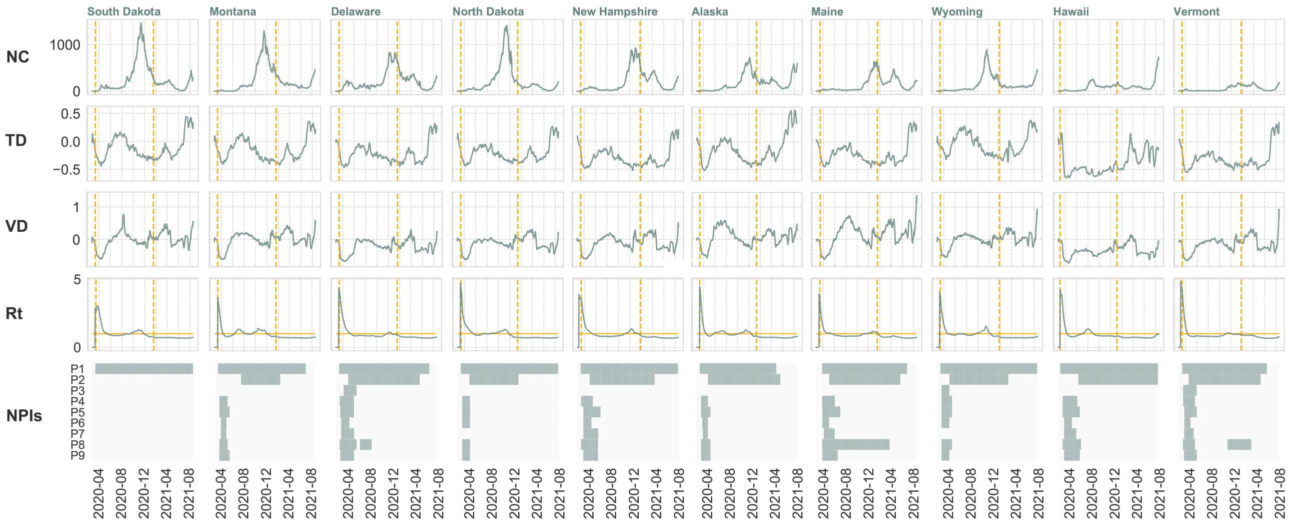

Supplementary Figure S5: Temporal changes in daily new COVID-19 cases (NC), travel distance difference (TD), visitation difference (VD), instantaneous reproduction number (R_t) and policy implementation in the ten states with 41th to 50th highest number of confirmed cases from Feb. 24 2020 to Aug. 18 2021.

5 SUPPLEMENTARY NOTE 5: TESTS FOR VAR MODELS

For clarity, we take the tests between R_t and *VD* as an example to illustrate in detail how the VAR and Toda-Yamamoto Granger causality tests are employed below. We first display the time series of variables selected (R_t, *VD*) in Supplementary Fig. S6. Here we employ a sub-period analysis to explore how variables and the relationships between them changed over time before and after the administration of the COVID-19 vaccines.

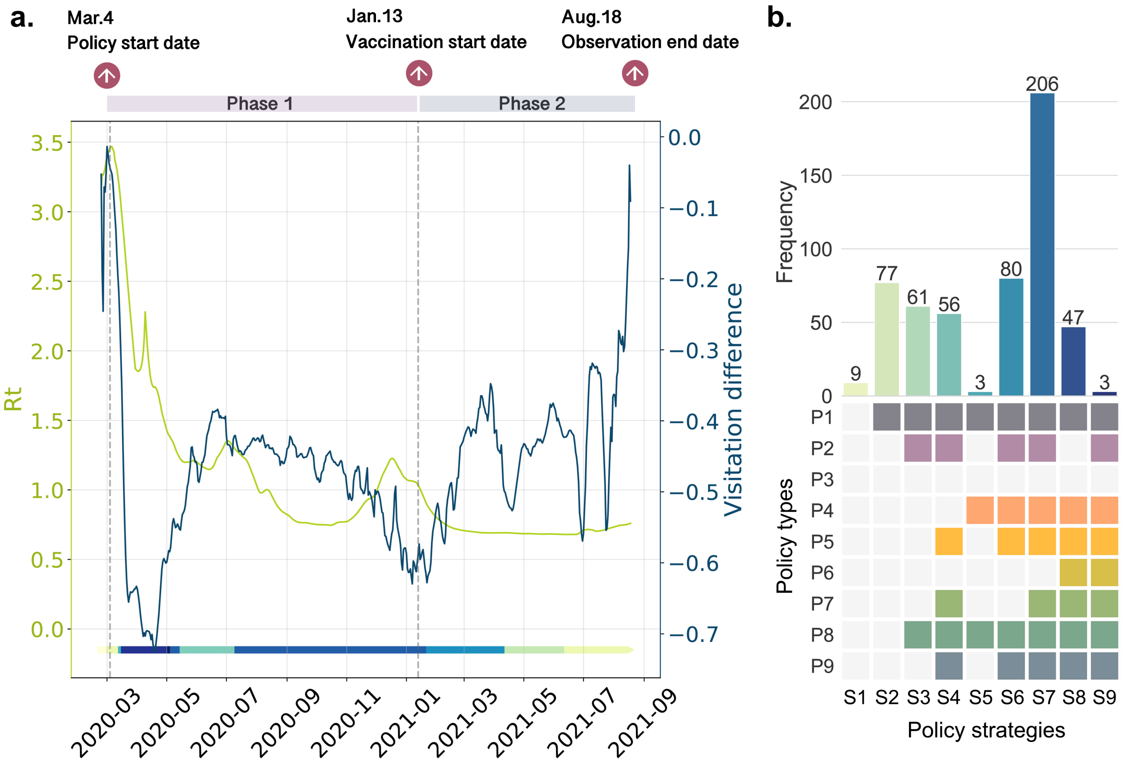

Supplementary Figure S6: Temporal changes in R_t, *VD*, and COVID-19 policy implementation in California during two phases. The start dates of policy implementation and vaccine distribution are indicated by dashed vertical lines in (**a**). Existing policy sets and their frequencies are displayed in (**b**). The temporal distribution of the policy sets is presented on the bottom of (**a**). The curves of R_t and *VD* are perceived as more tortuous in phase 1. R_t experienced a significant decrease from up to 3.469 to below 1 in phase 1. After the vaccines were released, *VD* roughly showed a rising trend and R_t, in contrast, declined gradually and kept steady below 1 with a slight increase displayed toward the end of observation. The strategies adopted during phase 1 are generally more diverse and stricter than those in phase 2.

We then perform unit root tests to assess whether the time series of R_t and *VD* are stationary and determine the order of integration of the variables. Here, to check the presence of a unit root, we adopt the Augmented Dickey-Fuller (ADF) test (Dickey and Fuller, 1979), for which the null hypothesis is that a unit root exists in the time series, amounting to a claim of non-stationarity. The maximum lags in the ADF are chosen according to a rule proposed by Schwert (Schwert, 2002). We also use the Phillips-Perron (PP) test (Phillips and Perron, 1988) to have a crosscheck. The results of the ADF test displayed in Supplementary Table S4 reveal that both variables R_t and VD are stationary in their levels at 1% significance level during phase 1. As for variables in phase 2, the unit root test results of R_t in level reject the null hypotheses, inferring that the time series is stationary. Meanwhile, the results of VD during phase 2 indicate the

presence of a unit root. In this case, we further apply the difference transformation to the time series of VD in phase 2, followed by performing the ADF test on the differenced series again. This time it is found to satisfy the stationarity condition on significance level 1%. PP tests give us the same results and thus confirmed our findings. The unit root test results signify that the variables of R_t and VD in phase 1, and R_t in phase 2 are individually integrated of order 0, denoted by $I(0)$. The time series of VD during phase 2 is stationary by differencing the series once, and thus integrated of the order $I(1)$.

	Variable	ADF		PP		Order of Integration
		t-Stat.	P-value	t-Stat.	P-value	
**Phase 1**	$\boldsymbol{R_t}$	-5.161***	0.0000	−5.871***	0.000	I(0)
	VD	−3.801***	0.0029	−3.937***	0.002	I(0)
**Phase 2**	$\boldsymbol{R_t}$	−4.725***	0.0001	−12.799***	0.000	I(0)
	VD	-0.752	0.8328	-0.904	0.787	I(1)
	**Δ*VD***	−3.802***	0.0029	−10.060***	0.000	-

Table S4. The results of the ADF and PP unit root tests on the selected time series variables R_t and *VD* in California. ***, **, and * indicate the rejection of the null hypothesis at the 1%, 5% and 10% significance levels, respectively. Δ denotes the first-differenced form of a variable.

What follows is the determination of the optimal lags m for the VAR systems by using four information criteria, the Akaike Information Criterion (AIC), Schwarz's Bayesian information criterion (SBIC), final prediction error (FPE), and Hannan–Quinn information criterion (HQIC). Here, the smaller the information criterion value, the better. As the results in Supplementary Table S5 show, the optimal lag lengths of $m = 2$ and $m = 9$ are chosen for phase 1 and phase 2 in California, respectively.

Lag	Phase 1 (Mar.4 2020 - Jan.12 2021)				Phase 2 (Jan.13 2021 - Aug.18 2021)			
	FPE	AIC	HQIC	SBIC	FPE	AIC	HQIC	SBIC
0	0.004	0.013	0.023	0.039	7.7×10^{-07}	-8.398	-8.384	-8.364
1	6.1×10^{-8}	-10.943	-10.912	-10.866	2.2×10^{-10}	-16.567	-16.525	-16.464
2	2.3×10^{-8}	-11.896	-11.844*	-11.767*	2.8×10^{-11}	-18.615	-18.545	-18.442*
3	2.3×10^{-8}	-11.898	-11.825	-11.716	2.7×10^{-11}	-18.649	-18.551	-18.407
4	2.4×10^{-8}	-11.882	-11.789	-11.649	2.5×10^{-11}	-18.725	-18.599	-18.414
5	2.4×10^{-8}	-11.861	-11.747	-11.576	2.5×10^{-11}	-18.730	-18.576	-18.350
6	2.5×10^{-8}	-11.838	-11.703	-11.501	2.5×10^{-11}	-18.730	-18.548	-18.281
7	2.5×10^{-8}	-11.849	-11.693	-11.460	2.3×10^{-11}	-18.810	-18.600	-18.292
8	2.2×10^{-8}	-11.938	-11.761	-11.498	2.2×10^{-11}	-18.874	-18.636*	-18.286
9	2.0×10^{-8}*	-12.031	-11.834	-11.539	2.2×10^{-11}*	-18.881*	-18.615	-18.225
10	2.0×10^{-8}	-12.030	-11.812	-11.487	2.2×10^{-11}	-18.872	-18.578	-18.146
11	2.0×10^{-8}	-12.031*	-11.793	-11.436	2.2×10^{-11}	-18.849	-18.527	-18.054
12	2.1×10^{-8}	-12.018	-11.759	-11.371	2.3×10^{-11}	-18.842	-18.492	-17.978

Table S5. Lag order selection for VAR models of R_t and *VD* during two phases in California under different criteria. * indicates the lag order selected by the criterion.

To check if the VAR models are well specified, we also conducted a series of tests, including whiteness test of residuals as shown in Supplementary Table S6 and stability test of the VAR systems in Supplementary Fig. S7. The estimation of VAR models and corresponding tests are performed using version 15.0 of STATA.

Lag	Phase 1 (Mar.4 2020 - Jan.12 2021)			Phase 2 (Jan.13 2021 - Aug.18 2021)		
	Chi-square	df	Prob. > Chi-square	Chi-square	df	Prob. > Chi-square
1	8.977	4	0.062	2.001	4	0.735
2	3.957	4	0.412	2.861	4	0.581

Table S6. Lagrange-multiplier test in estimated VAR models for R_t and *VD* during two phases in California. H0: no autocorrelation at lag order.

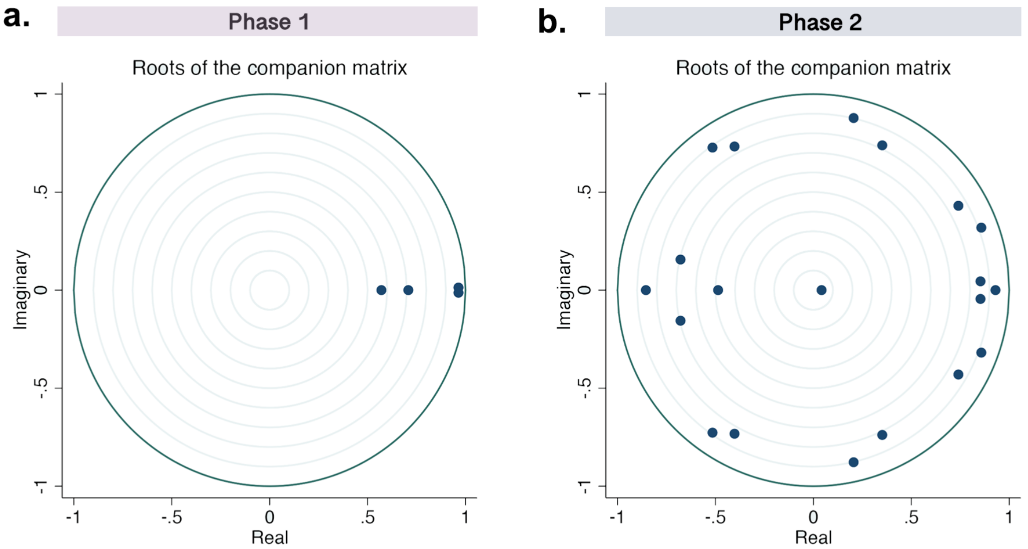

Supplementary Figure S7: Eigenvalue stability condition for the estimated VAR models for R_t and *VD* during two phases in California. All the eigenvalues lie inside the unit circle. Each VAR satisfies stability condition.

6 SUPPLEMENTARY NOTE 6: ADDITIONAL TODA AND YAMAMOTO CAUSALITY TEST RESULTS

To introduce the idea of Granger causality more concretely, let $\boldsymbol{x} = [x_1, x_2, \ldots, x_n]$ and $\boldsymbol{y} = [y_1, y_2, \ldots, y_n]$ denote the two stationary time series of length n. To examine whether time series $\boldsymbol{x}$ Granger causes $\boldsymbol{y}$, consider the following model:

$$y_t = \gamma + \sum_{i=1}^{m} \alpha_i y_{t-i} + \sum_{i=1}^{m} \phi_i x_{t-i} + \varepsilon_t \tag{S9}$$

where y_t denotes the value of the time series $\boldsymbol{y}$ at time t, i is the length of the lag-time moving window, α_i and ϕ_i are the parameters to estimate, ϵ_t refers to the white noise residual. In this setting, the Granger causality can be investigated based on an F test with the null hypothesis of $H_0 : \phi_1 = \phi_2 = \ldots = \phi_i = 0$. Essentially, if H_0 is rejected, one can conclude that the Granger causality from time series $\boldsymbol{x}$ to $\boldsymbol{y}$ exists, since that the lagged values of $\boldsymbol{x}$ provide additional explanatory and predictive power to the regression model. Based on the conventional Granger causality test introduced above, Toda and Yamamoto (Toda and Yamamoto, 1995) proposed a modified version that overcomes several limitations of traditional approaches in hypothesis testing when there are unit roots in the VAR system. Specifically, Toda and Yamamoto causality test does not require pre-testing for the cointegrating properties of the system, and thus avoids the potential bias associated with unit roots and cointegration tests (Clarke and Mirza, 2006; Zapata and

Rambaldi, 1997). Given these advantages of the Toda and Yamamoto causality test, we adopt this method in the present study.

Supplementary Table S7 presents the results of Granger causality tests between the variable pairs of (R_t, VD) in ten states affected the most by coronavirus in the U.S.

State	Direction	Phase 1				Phase 2			
		Lag(m)	Lag(m+d)	Chi-square	Prob.	Lag(m)	Lag(m+d)	Chi-square	Prob.
CA	$R_t \to VD$	2	2	16.086^{***}	0.000	9	10	11.680	0.307
	$VD \to R_t$	2	2	2.531	0.282	9	10	16.209^{*}	0.094
TX	$R_t \to VD$	2	3	15.046^{***}	0.002	12	13	42.174^{***}	0.000
	$VD \to R_t$	2	3	9.585^{**}	0.022	12	13	9.146	0.762
FL	$R_t \to VD$	12	13	38.877^{***}	0.000	3	4	2.065	0.724
	$VD \to R_t$	12	13	14.379	0.348	3	4	10.466^{**}	0.033
NY	$R_t \to VD$	3	4	5.903	0.207	7	8	15.674^{**}	0.047
	$VD \to R_t$	3	4	16.070^{***}	0.003	7	8	51.799^{***}	0.000
IL	$R_t \to VD$	2	3	13.937^{***}	0.003	8	9	19.109^{**}	0.024
	$VD \to R_t$	2	3	1.771	0.621	8	9	13.983	0.123
PA	$R_t \to VD$	3	4	15.439^{***}	0.004	8	9	8.766	0.024
	$VD \to R_t$	3	4	0.423	0.981	8	9	24.810^{***}	0.003
GA	$R_t \to VD$	2	3	13.570^{***}	0.004	7	8	3.171	0.923
	$VD \to R_t$	2	3	4.302	0.231	7	8	15.465^{*}	0.051
OH	$R_t \to VD$	8	9	9.450	0.397	7	8	12.822	0.118
	$VD \to R_t$	8	9	21.102^{**}	0.012	7	8	22.556^{***}	0.004
NC	$R_t \to VD$	8	9	12.647	0.179	8	9	4.129	0.903
	$VD \to R_t$	8	9	21.741^{**}	0.010	8	9	10.847	0.286
NJ	$R_t \to VD$	3	4	6.543	0.162	8	9	6.119	0.728
	$VD \to R_t$	3	4	1.225	0.874	8	9	17.521^{**}	0.041

Table S7. Toda and Yamamoto causality test results for R_t and VD pairs in the ten states most affected by the coronavirus in the U.S.. ***, **, and * indicate the rejection of the null hypothesis at the 1%, 5% and 10% significance levels, respectively.

7 SUPPLEMENTARY NOTE 7: EXISTING OPTIMAL POLICY STRATEGIES FOR STATES

The concept of Pareto efficiency Pareto (1964) is originally introduced to describe an economic state in which the reallocation of resources cannot make at least one person better off without making any other individual worse off Fudenberg and Tirole (1991). We evaluate the performance of existing policy strategies during two phases across ten states with the highest number of COVID-19 cases using Pareto analysis in Supplementary Table S8 and Table S9.

State	Mean R_t	Mean v_t	Policy types									Strategy	pct (%)
			P1	P2	P3	P4	P5	P6	P7	P8	P9		
CA	1.206	-0.319	1	1	0	0	1	0	1	1	1	1	5
	0.769	-0.393	1	1	0	1	1	0	1	1	1	2	5
TX	0.789	-0.187	1	1	0	0	0	0	0	1	0	3	25
	0.880	0.483	1	1	0	0	0	0	0	0	0	4	30
FL	0.757	-0.169	1	1	0	0	0	0	0	1	0	3	25
	0.805	-0.132	1	1	0	0	0	0	0	0	0	4	30
NY	0.692	-0.217	1	1	0	0	1	0	0	1	1	5	5
	0.961	0.160	1	1	0	0	1	0	0	1	0	6	15
IL	0.768	-0.197	1	1	0	0	1	0	0	1	0	6	15
	0.782	-0.16	1	1	0	0	0	0	0	0	0	4	30
	0.792	-0.031	1	1	0	1	1	0	0	1	0	7	5
PA	0.767	-0.117	1	1	0	0	0	0	0	1	0	3	25
	0.952	0.159	1	1	0	1	1	0	0	1	1	8	5
	0.841	-0.023	1	1	0	0	0	0	0	1	0	3	25
GA	0.822	0.047	1	1	0	0	0	0	0	0	0	4	30
OH	0.894	0.065	1	1	0	0	0	0	0	0	0	4	30
NC	0.851	-0.076	1	1	0	0	1	0	0	1	0	6	15
	0.935	0.153	1	1	0	0	0	0	0	1	0	3	25
NJ	0.692	-0.146	1	1	0	0	1	0	0	0	1	9	5
	0.844	0.257	1	1	0	0	0	0	0	0	0	4	30
pct of a policy type (%)			100	100	0	15	45	0	10	65	25	-	-

Table S8. Existing optimal policy strategies for the ten states most affected by COVID-19 during phase 1.

State	Mean R_t	Mean v_t	Policy types									Strategy	pct (%)
			P1	P2	P3	P4	P5	P6	P7	P8	P9		
CA	0.694	-0.254	1	1	0	1	1	0	0	1	1	1	5
	0.681	-0.369	1	1	0	0	0	0	0	1	0	2	18
	0.693	-0.311	1	0	0	0	0	0	0	0	0	3	27
TX	0.693	-0.150	1	0	0	0	0	0	0	0	0	3	27
FL	0.758	0.172	1	1	0	0	0	0	0	0	0	4	27
	0.659	-0.362	1	0	0	0	0	0	0	0	0	3	27
NY	0.761	0.319	1	1	0	0	0	0	0	1	0	2	18
	0.694	-0.228	0	0	0	0	0	0	0	0	0	5	23
IL	0.702	0.011	1	1	0	0	0	0	0	0	0	4	27
	0.682	-0.374	1	0	0	0	0	0	0	0	0	3	27
PA	0.733	0.308	1	1	0	0	0	0	0	1	0	2	18
	0.691	-0.113	0	0	0	0	0	0	0	0	0	5	23
GA	0.709	0.379	1	1	0	0	0	0	0	0	0	4	27
	0.683	-0.421	1	0	0	0	0	0	0	0	0	3	27
	0.683	-0.371	0	0	0	0	0	0	0	0	0	5	23
OH	0.709	0.405	1	1	0	0	0	0	0	0	0	4	27
	0.686	-0.122	0	0	0	0	0	0	0	0	0	5	23
NC	0.717	0.544	1	1	0	0	0	0	0	1	0	2	18
	0.716	0.176	1	1	0	0	0	0	0	0	0	4	27
	0.682	-0.244	1	0	0	0	0	0	0	0	0	3	27
NJ	0.774	0.193	1	1	0	0	0	0	0	0	0	4	27
	0.693	-0.182	0	0	0	0	0	0	0	0	0	5	23
pct of a policy type (%)			77	50	0	5	5	0	0	23	5	-	-

Table S9. Existing optimal policy strategies for the ten states most affected by COVID-19 during phase 2.

8 SUPPLEMENTARY NOTE 8: POTENTIAL OPTIMAL POLICY STRATEGIES CREATED BY NSGA-II ALGORITHM

To generate new policy strategies for each individual state, we employ the NSGA-II algorithm Deb et al. (2002) by using the Platypus package Hadka (2019) for multi-objective evolutionary computing in Python 3.7. NSGA-II differs from traditional genetic algorithms in two aspects: (1) the selection of appropriate individual solutions among possible ones to generate the next generation is based on their dominance levels according to the Pareto optimality; (2) The crowding distance is employed as a measure to make choices between individual solutions that have the same dominance level. Additionally, NSGA-II adopts an elitist strategy for the selection that unless better solutions are found, the best ones obtained so far are retained. The algorithm produces a set of optimal solutions that collectively make up the Pareto front when the optimization process is terminated Yoo and Harman (2010).

We employ NSGA-II algorithm to generate top ten new optimal policy strategies for each state during different phases as shown in Supplementary Fig. S8-S16. Strategies are listed in ascending order of the predicted $\hat{R}_t$. If less than ten optimal solutions are distilled for a certain state and phase, we list all the optimal solutions.

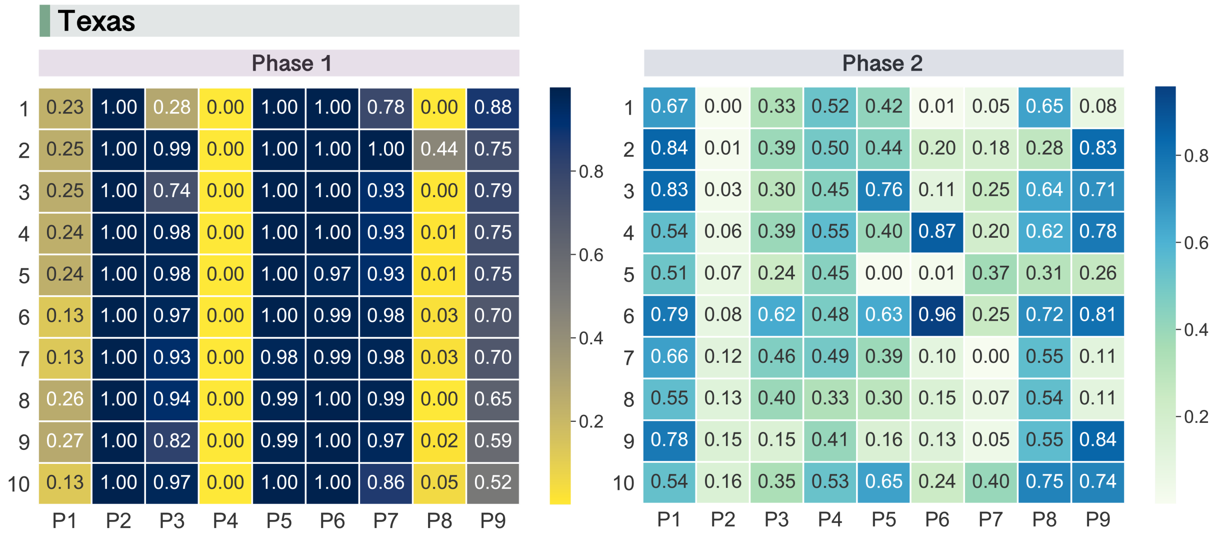

Supplementary Figure S8: Top ten potential Pareto optimal policy strategies generated for Texas.

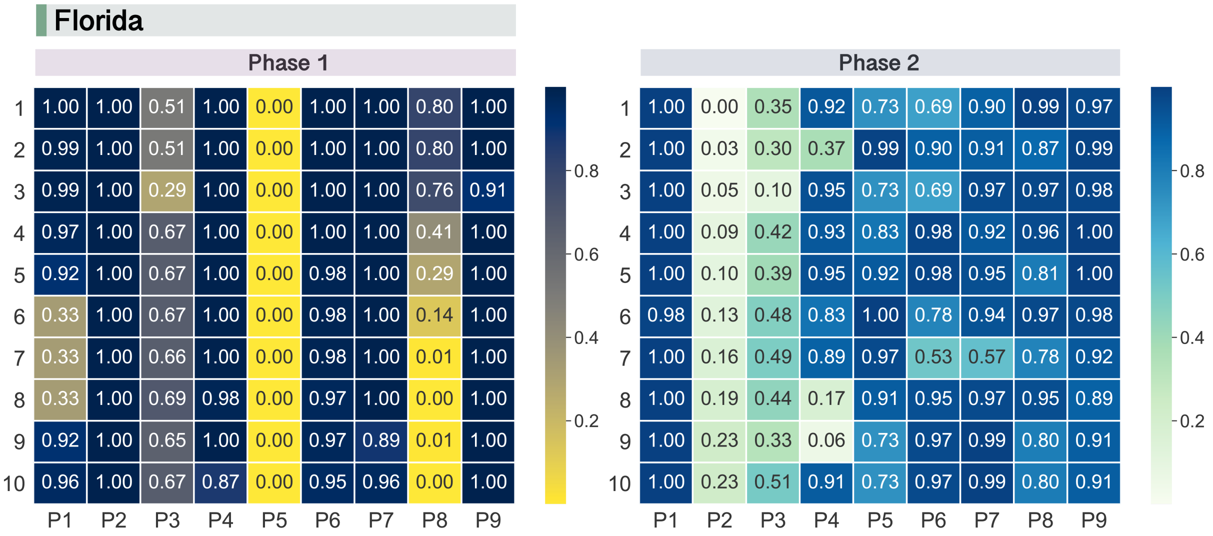

Supplementary Figure S9: Top ten potential Pareto optimal policy strategies generated for Florida.

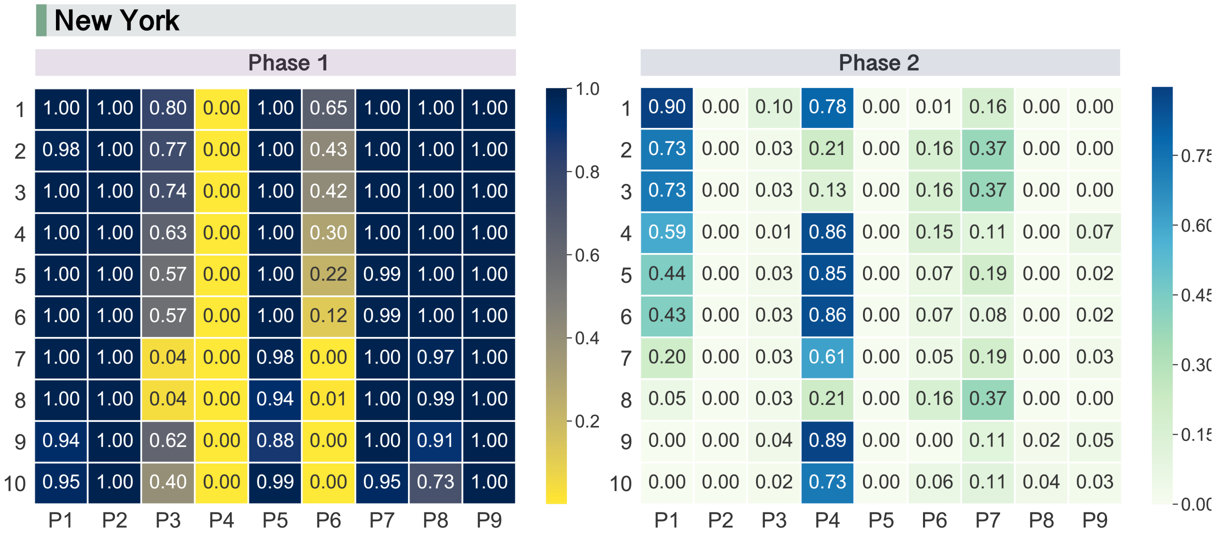

Supplementary Figure S10: Top ten potential Pareto optimal policy strategies generated for New York.

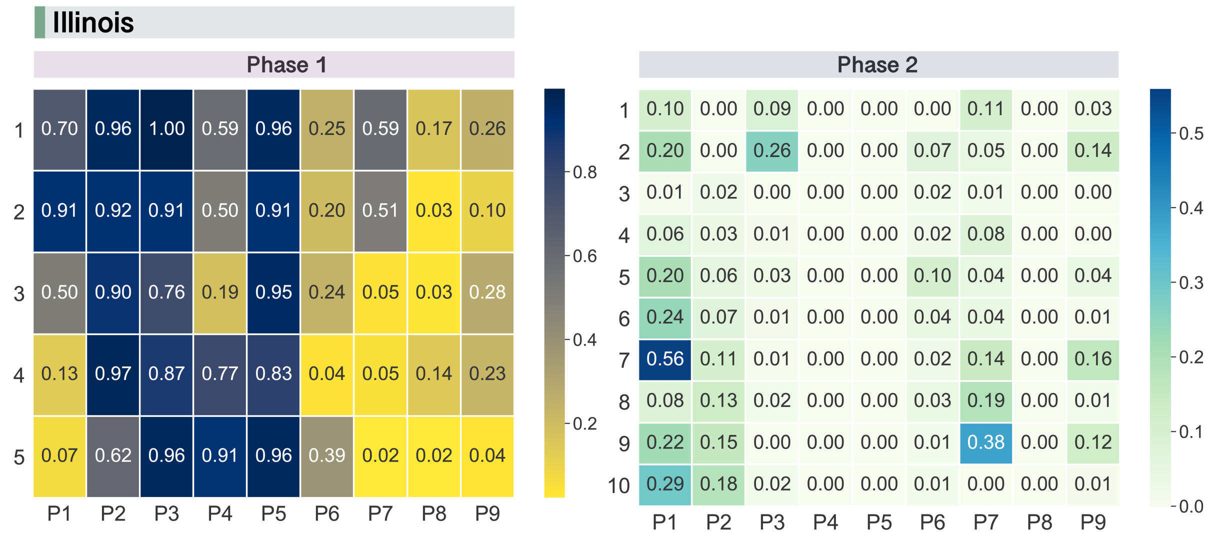

Supplementary Figure S11: Top potential Pareto optimal policy strategies generated for Illinois.

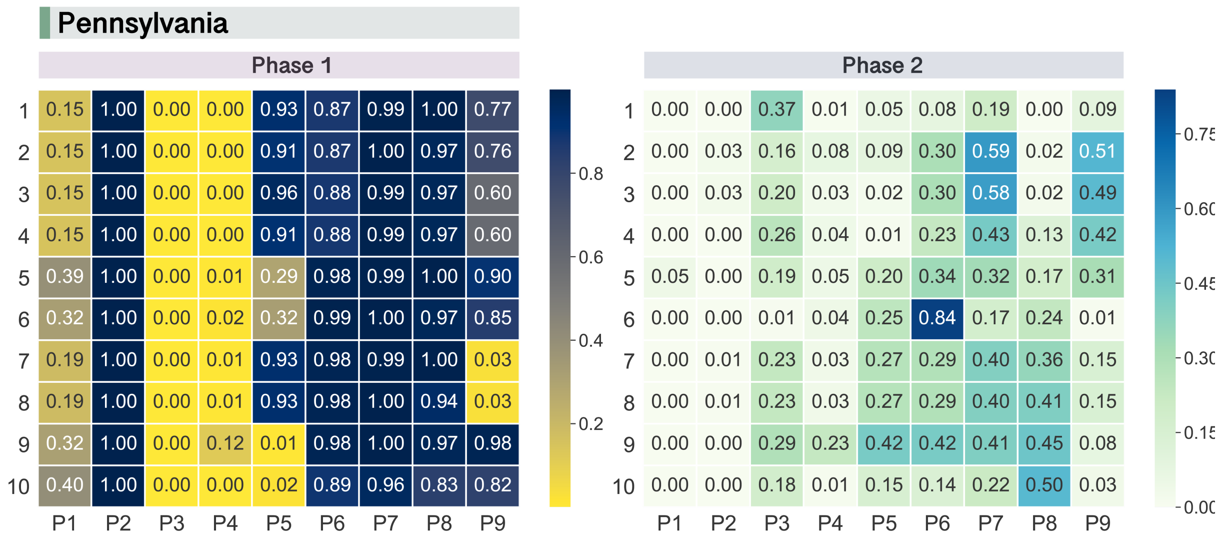

Supplementary Figure S12: Top ten potential Pareto optimal policy strategies generated for Pennsylvania.

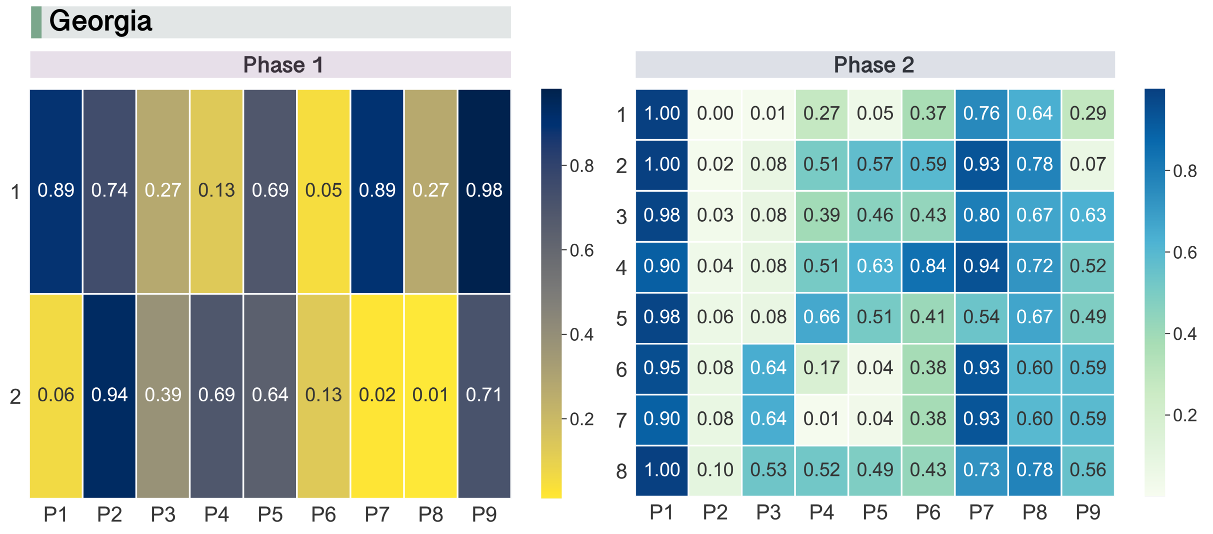

Supplementary Figure S13: Top potential Pareto optimal policy strategies generated for Georgia.

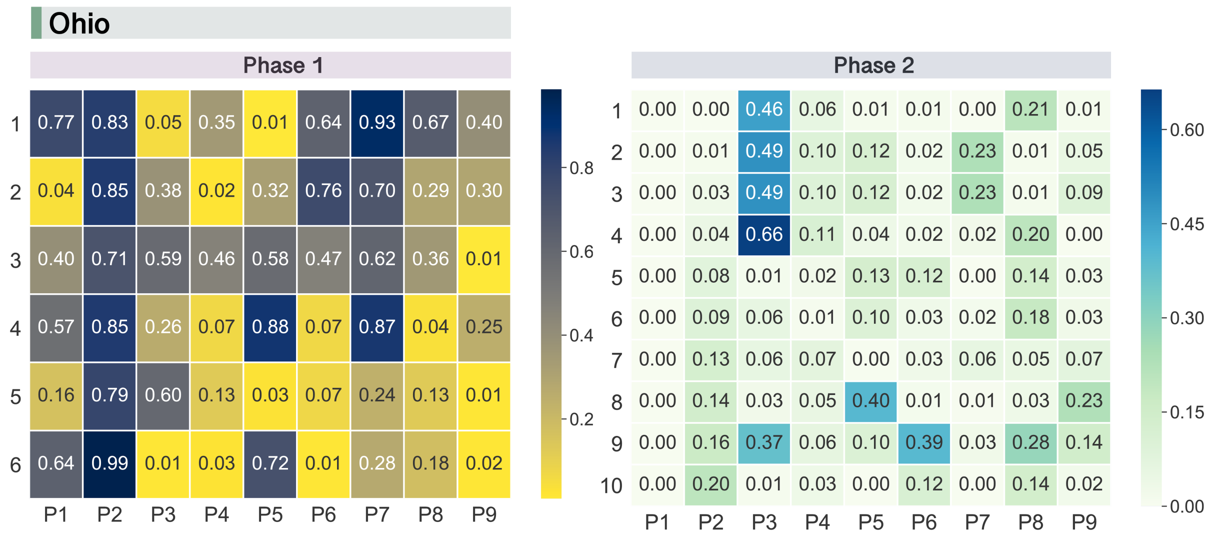

Supplementary Figure S14: Top potential Pareto optimal policy strategies generated for Ohio.

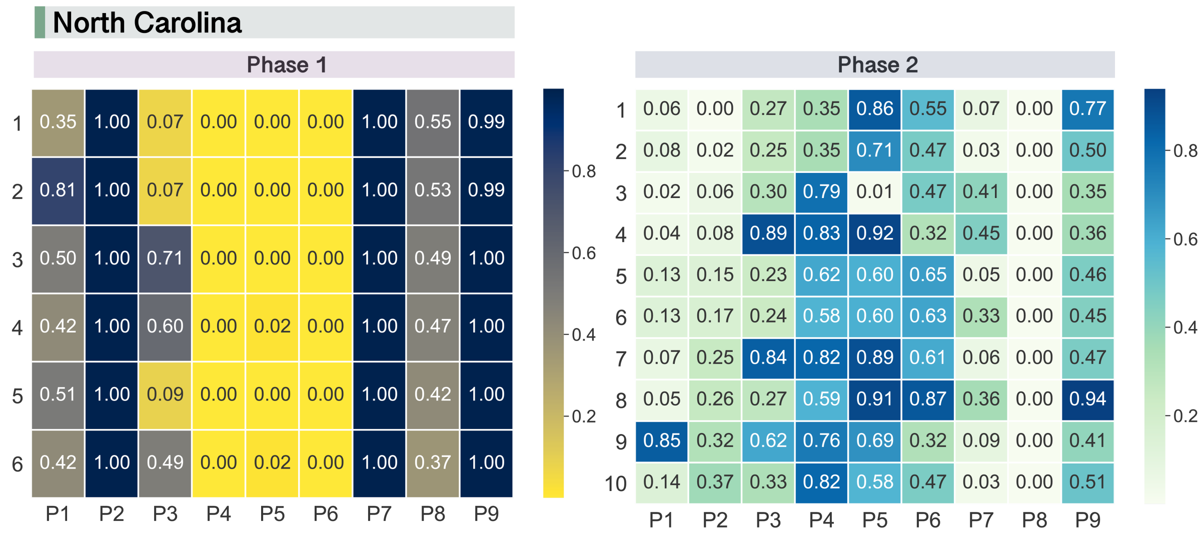

Supplementary Figure S15: Top potential Pareto optimal policy strategies generated for North Carolina.

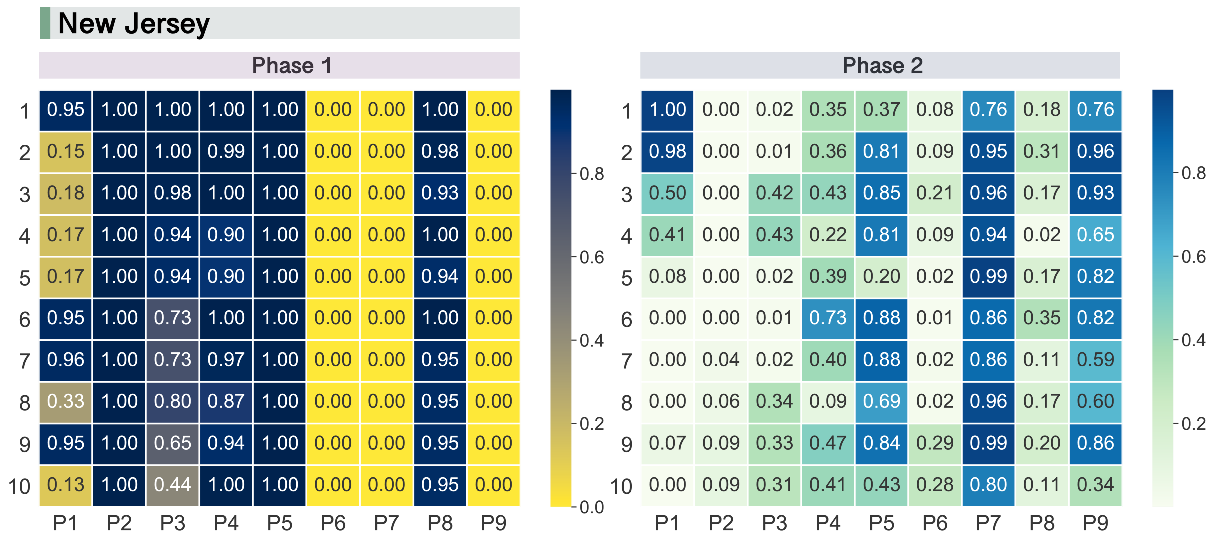

Supplementary Figure S16: Top ten potential Pareto optimal policy strategies generated for New Jersey.

9 SUPPLEMENTARY NOTE 9: SENSITIVITY ANALYSIS OF THE METHODOLOGY

To account for the multiple variants of COVID-19, which can lead to variations in epidemic parameters, we conducted sensitivity analyses to test the robustness of our primary findings and conclusions. However, obtaining granular data at the state level regarding the prevalence of specific virus variants during specific time periods is not feasible. Additionally, virus variants do not exist exclusively within specific time frames; they often coexist simultaneously. Therefore, determining the exact combination of COVID-19 variants per day during the observation period in each state is not possible. Instead, we focused on the primary variants during the study period in the U.S. and their corresponding epidemic parameters. By modifying the initial hyperparameters in the epidemic model, we tested the sensitivity of our methodology and assessed any potential impact on the main results.

According to Our World in Data (OWD, 2021), the primary variants in the U.S. during our research period from February 24, 2020, to August 18, 2021, were the Alpha (B.1.1.7) and Delta (B.1.617.2) variants. We obtained the transmission parameters for these variants from relevant literature (Galmiche et al., 2023) and incorporated them into our model. The results aligned with the VAR analysis, Granger causality tests, and optimized policy conclusions mentioned in the main text. For instance, taking California as an example, using the parameters for the Alpha (B.1.1.7) variant (4.96 days [95% CI 4.90–5.02]) and the Delta (B.1.617.2) variant (4.43 days [95% CI 4.36–4.49]), the results of the Granger causality test are shown in Supplementary Table S10 and Supplementary Table S11. As evident from the results, the statistical test results of the Toda-Yamamoto Granger causality are generally consistent with Table 1 in the main text, indicating a similar pattern across different COVID-19 variants. While the number of lag days may vary for different variants during phase 2, the statistically significant levels remain consistent. However, due to space constraints, we have not included the complete set of results in this paper. For readers who are interested, we provide access to the data and code associated with the paper, allowing them to conduct further experiments and explore additional findings.

Direction	Phase 1 (Mar.4 2020 - Jan.12 2021)				Phase 2 (Jan.13 2021 - Aug.18 2021)			
	Lag(m)	Lag(m+d)	Chi-square	Prob.	Lag(m)	Lag(m+d)	Chi-square	Prob.
$R_t \to VD$	2	2	15.980^{***}	0.000	9	10	11.603	0.312
$VD \to R_t$	2	2	1.515	0.469	9	10	16.208^{*}	0.094

Table S10. Toda-Yamamoto Granger causality test results for transmission and mobility variables in California fitted by Alpha (B.1.1.7) variant (4.96 days [95% CI 4.90–5.02]). ***, **, and * indicate the rejection of the null hypothesis at the 1%, 5% and 10% significance levels, respectively.

Direction	Phase 1 (Mar.4 2020 - Jan.12 2021)				Phase 2 (Jan.13 2021 - Aug.18 2021)			
	Lag(m)	Lag(m+d)	Chi-square	Prob.	Lag(m)	Lag(m+d)	Chi-square	Prob.
$R_t \to VD$	2	2	16.844^{***}	0.000	2	3	2.269	0.518
$VD \to R_t$	2	2	3.396	0.183	2	3	7.545^{*}	0.056

Table S11. Toda-Yamamoto Granger causality test results for transmission and mobility variables in California fitted by Delta (B.1.617.2) variant (4.43 days [95% CI 4.36–4.49]). ***, **, and * indicate the rejection of the null hypothesis at the 1%, 5% and 10% significance levels, respectively.

REFERENCES

[Dataset] (2021). The new york times. coronavirus (covid-19) data in the united states. https://github.com/nytimes/covid-19-data. Accessed: 2021-08-27

[Dataset] (2021). Our world in data. sars-cov-2 sequences by variant. https://ourworldindata.org/grapher/covid-variants-bar?time=2021-08-16. Accessed: 2023-06-12

Bjørnstad, O. N., Shea, K., Krzywinski, M., and Altman, N. (2020). The seirs model for infectious disease dynamics. *Nature Methods* 17, 557–559

Clarke, J. A. and Mirza, S. (2006). A comparison of some common methods for detecting granger noncausality. *Journal of Statistical Computation and Simulation* 76, 207–231

Deb, K., Pratap, A., Agarwal, S., and Meyarivan, T. (2002). A fast and elitist multiobjective genetic algorithm: Nsga-ii. *IEEE transactions on evolutionary computation* 6, 182–197

Dickey, D. A. and Fuller, W. A. (1979). Distribution of the estimators for autoregressive time series with a unit root. *Journal of the American statistical association* 74, 427–431

Fudenberg, D. and Tirole, J. (1991). *Game Theory* (MIT Press)

Galmiche, S., Cortier, T., Charmet, T., Schaeffer, L., Chény, O., von Platen, C., et al. (2023). Sars-cov-2 incubation period across variants of concern, individual factors, and circumstances of infection in france: a case series analysis from the comcor study. *The Lancet Microbe*

[Dataset] Hadka, D. (2019). Platypus-multiobjective optimization in python (2015)

Li, Q., Guan, X., Wu, P., Wang, X., Zhou, L., Tong, Y., et al. (2020). Early transmission dynamics in wuhan, china, of novel coronavirus–infected pneumonia. *New England Journal of Medicine*

Maier, B. F. and Brockmann, D. (2020). Effective containment explains subexponential growth in recent confirmed covid-19 cases in china. *Science* 368, 742–746

Pareto, V. (1964). *Cours d'économie politique*, vol. 1 (Librairie Droz)

Phillips, P. C. and Perron, P. (1988). Testing for a unit root in time series regression. *Biometrika* 75, 335–346

[Dataset] Raifman, J., Nocka, K., Jones, D., Bor, J., Lipson, S., Jay, J., et al. (2020). Covid-19 us state policy database. www.tinyurl.com/statepolicies

Schwert, G. W. (2002). Tests for unit roots: A monte carlo investigation. *Journal of Business & Economic Statistics* 20, 5–17

Seow, J., Graham, C., Merrick, B., Acors, S., Steel, K. J., Hemmings, O., et al. (2020). Longitudinal evaluation and decline of antibody responses in sars-cov-2 infection. *MedRxiv*

Toda, H. Y. and Yamamoto, T. (1995). Statistical inference in vector autoregressions with possibly integrated processes. *Journal of econometrics* 66, 225–250

Verity, R., Okell, L. C., Dorigatti, I., Winskill, P., Whittaker, C., Imai, N., et al. (2020). Estimates of the severity of coronavirus disease 2019: a model-based analysis. *The Lancet infectious diseases*

Yoo, S. and Harman, M. (2010). Using hybrid algorithm for pareto efficient multi-objective test suite minimisation. *Journal of Systems and Software* 83, 689–701

Zapata, H. O. and Rambaldi, A. N. (1997). Monte carlo evidence on cointegration and causation. *Oxford Bulletin of Economics and statistics* 59, 285–298
